# Effectiveness of Prenyl Group on Flavonoids from *Epimedium koreanum* Nakai on Bacterial Neuraminidase Inhibition

**DOI:** 10.3390/molecules24020317

**Published:** 2019-01-16

**Authors:** Hong Min Choi, Jeong Yoon Kim, Zuo Peng Li, Janar Jenis, Yeong Jun Ban, Aizhamal Baiseitova, Ki Hun Park

**Affiliations:** Division of Applied Life Science (BK21 plus), IALS, Gyeongsang National University, Jinju 52828, Korea; sgiantm@naver.com (H.M.C.); foryou6633@gmail.com (J.Y.K.); dameng@126.com (Z.P.L.); janarjenis@mail.ru (J.J.); banyoung972@naver.com (Y.J.B.); aizhabaiseitova@gmail.com (A.B.)

**Keywords:** *Epimedium koreanum* Nakai, koreanoside F, koreanoside G, bacterial neuraminidase, binding affinity

## Abstract

In this study, the inhibitory potential of bacterial neuraminidase (NA) was observed on the leaves of *Epimedium koreanum* Nakai, which is a popular ingredient in traditional herbal medicine. This study attempted to isolate the relevant, responsible metabolites and elucidate their inhibition mechanism. The methanol extraction process yielded eight flavonoids (**1**–**8**), of which compounds **7** and **8** were new compounds named koreanoside F and koreanoside G, respectively. All the compounds (**1**–**8**) showed a significant inhibition to bacterial NA with IC_50_ values of 0.17–106.3 µM. In particular, the prenyl group on the flavonoids played a critical role in bacterial NA inhibition. Epimedokoreanin B (compound **1**, IC_50_ = 0.17 µM) with two prenyl groups on C8 and C5′ of luteolin was 500 times more effective than luteolin (IC_50_ = 85.6 µM). A similar trend was observed on compound **2** (IC_50_ = 0.68 µM) versus dihydrokaempferol (IC_50_ = 500.4 µM) and compound **3** (IC_50_ = 12.6 µM) versus apigenin (IC_50_ = 107.5 µM). Kinetic parameters (*K*_m_, *V*_max_, and *K*_ik_/*K*_iv_) evaluated that all the compounds apart from compound **5** showed noncompetitive inhibition. Compound **5** was proven to be a mixed type inhibitor. In an enzyme binding affinity experiment using fluorescence, affinity constants (*K*_SV_) were tightly related to inhibitory activities.

## 1. Introduction

The neuraminidases (EC 3.2.1.18) are enzymes that catalyze the hydrolysis of terminal neuraminic acid from a variety of glycoproteins and gangliosides. Bacterial neuraminidase (NA) preferentially cleaves 5-*N*-acetylneuraminic acid (Neu5Ac) from cell membrane glycoproteins via the linkage between neuraminic acid and α→3 or α2→6 galactose [1,2,3]. Bacterial neuraminidase is expressed among particular pathogens, such as *Streptococcus pneumonia*, *Pseudomonas aeruginosa*, and *Clostridium perfringens*, and it cleaves neuraminic acid from glycoconjugates. The key function of bacterial NA is to help promote mucosal infection via biofilm formation [4]. Chen et al. reported that bacterial NA inhibition blocks the desialylation of CD24 that occurring in sepsis, which preserves the Siglec inhibitory circuit and suppresses the inflammatory response. At the same time, bacterial NA acts with bacterial virulence, implicating biofilms formation and bacterial defense against antibiotics [5]. Consequently, bacterial NA implicated in numerous pathways involved in bacterial infection and its inhibitors will provide one of the ways to treat bacterial pathogenic diseases depending on the neuraminic acid hydrolysis [6]. Previously, our group has succeeded in isolating and characterizing the naturally occurring neuraminidase inhibitors, which are xanthones, pterocarpans, and geranylated flavonoids from *Garcinia mangostana* [7], *Sophora flavescens* [8], and *Paulownia tomentosa* [9].

*Epimedium koreanum* Nakai belongs to the *Berberidaceae* family and has a unique feature, having three branches and three leaves on each branch. It grows in Southeast Asian countries [10]. The aboveground parts of *E. koreanum* Nakai (leaves and stem) have been used as a medicinal herb for a general tonic against infertility, as well as against inflammatory diseases including cardiovascular diseases and arthritis [11,12]. Nowadays, the leaves are consumed as a popular medicinal herb. Its species continues to be a rich source of phenolic metabolites, of which prenylated flavonoids are the major constituents. Based on the composition of the phenolic metabolites, they display a broad spectrum of biological activities, such as antioxidative, anticancer, immunomodulatory, and neuroprotective functions [13,14].

In this study, we isolated eight prenylated flavonoids from using a methanol extraction process on the leaves of *E. koreanum* Nakai, and their structures were fully characterized by spectroscopic methods. All the isolated compounds were examined for bacterial NA inhibition and kinetic behavior. In particular, we observed a critical role of the prenyl group on the flavonoids in enzyme inhibition.

## 2. Results and Discussion

### 2.1. Isolation of Flavonoids from E. koreanum Nakai

**Figure 1:**
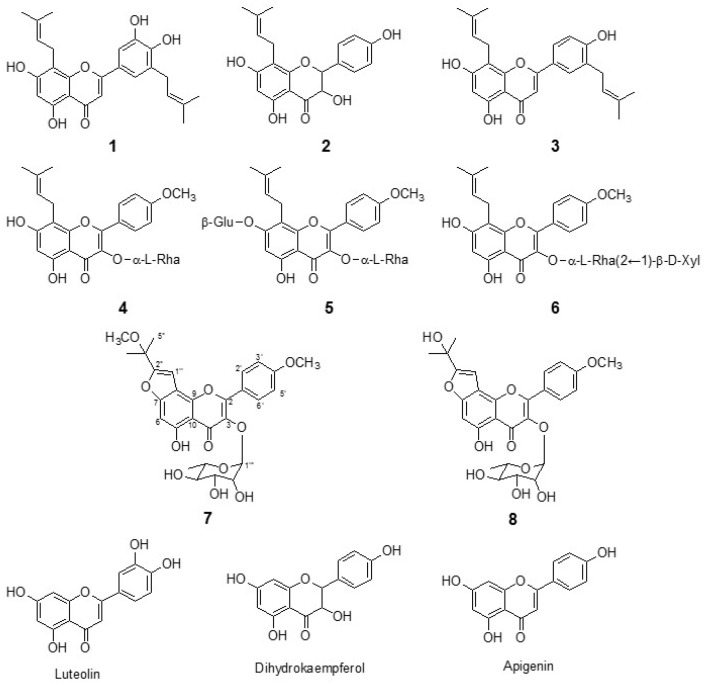
Chemical structures of flavonoids (**1**–**8**) from *Epimedium koreanum* Nakai.

In the preliminary screening, we observed that the ethyl acetate fraction of the methanol extract of *E. koreanum* Nakai leaves showed potent inhibition (80% inhibition at 50 μg/mL) of bacterial neuraminidase (NA). The ethyl acetate fractions were purified over silica gel, C18 reversed-phase silica gel, and Sephadex LH-20 as described in Section 3.1 to find out the compounds responsible for the bacterial NA inhibition. The isolated compounds were identified as known prenylated flavonoids (**1**–**6**) and two new flavonoids, compounds **7** and **8**. As shown in Figure 1, the flavonoids (compounds **1**–**6**) were identified as epimedokoreanin B (compound **1**), 8-(γ,γ-dimethyl allyl)-5,7,4′-trihydroxydihydroflavonol (compound **2**), 5,7,4′-trihydroxy-8,3′-diprenyl flavone (compound **3**), icariside II (compound **4**), icariin (compound **5**), and sagittatoside B (compound **6**).

**Figure 2:**
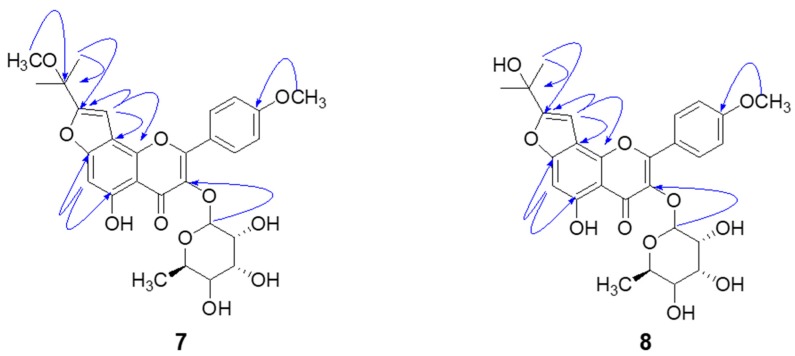
HMBC correlation (H→C) of the new compounds **7** and **8**.

Compound **7** was isolated as a yellow powder with the molecular formula C_28_H_31_O_11_ by the [M + H]^+^ ion at 543.1906 (Calcd 543.1788) in HRFABMS. ^1^H and ^13^C-NMR data in conjunction with DEPT experiments indicated the presence of 28 carbons consisting of the following functional groups: 6 methines (sp^2^), 5 methines (sp^3^), 5 methyls, and 12 quaternary carbons (Table 1). The analysis of 14 degrees of unsaturation indicated pentacyclic skeleton for compound **7**. A typical flavonol skeleton was deduced by C2 (*δ*c 157.8), C3 (*δ*c 135.9), and α, β-unsaturated carbonyl group (*δ*c 179.1). One set of A_2_B_2_ resonance at *δ*_H_ 7.04 (2H, d, *J* = 8.7 Hz) and *δ*_H_ = 7.88 (2H, d, *J* = 8.7 Hz) indicated the presence of a para substituted ring B. A strong HMBC correlation between 4′-OCH_3_ (*δ*_H_ 3.81) and C-4′ (*δ*c 162.3) confirmed the location of the methoxy group. The prenyl-derived side chain was unveiled to be a 2-methoxydimethyl-furan moiety by the HMBC correlation of H-1″ (*δ*_H_ 6.93) with C-2″ (*δ*c 159.1), as well as CH_3_ (C5″, *δ*_H_ 3.03) with C-3″ (*δ*c 73.3) and C-2″ (*δ*c 159.1). The methoxy group on C-3″ was confirmed with a strong HMBC between 3″-CH_3_O (*δ*_H_ 3.03) and C-3″. The furan moiety was placed on C7 and C8, because H-1″ (*δ*_H_ 6.93, s) had a HMBC correlation with C-8 (*δ*c 108.9) and C-9 (*δ*c 158.2). Additionally, H-6 (*δ*_H_ 6.78, s) was confirmed by the HMBC correlation of H-6 with C-5 (*δ*c 159.5) and C-7 (*δ*c 148.9). The rhamnose (Rha) moiety was clearly confirmed with CH_3_ (*δ*_H_ 0.82, 3H, d, *J* = 5.8 Hz), H-1 (*δ*_H_ 5.38, s), H-2 (*δ*_H_ 3.19, m), H-3 (*δ*_H_ 4.16, m), H-4 (*δ*_H_ 3.63, m), H-5 (*δ*_H_ 3.25, m), and H-6 (*δ*_H_ 0.82, d, *J* = 5.8 Hz). A clear HMBC correlation of anomeric H (*δ*_H_ 5.38) and C-3 (*δ*c 135.9) indicated that rhamnose connected to C-3. Simultaneously, the α-configuration at the anomeric carbon was assigned on the basis of the coupling constant (*J* < 1 Hz) (Table 1 and Figure 2). Thus, compound **7** was determined to be 5-hydroxy-2-(4-methoxyphenyl)-8-(2-methoxypropan-2-yl)-3-(((2S,3R,4R,5R,6S)-3,4,5-trihydroxy-6-methyltetra-hydro-2H-pyran-2-yl)oxy)-4H-furo[2-3-h]chromen-4-one, named koreanoside F.

Compound **8** was a yellow powder having molecular formula C_27_H_28_O_11_ and 14 degrees of unsaturation [HRFABMS (*m*/*z* 529.1682 [M + H]^+^, Calcd 529.1632)]. The ^1^H and ^13^C-NMR data of compound **8**, fully assigned through 2D NMR experiments, closely resembled those of compound **7** (Table 1). Given the broad spectral similarities between this species and compound **7**, we focused on identifying the furan moiety therein. The hydroxydimethyl group on the furan ring was confirmed by the HMBC correlation of CH_3_ (C5″, *δ*_H_ 1.66) with C-3″ (*δ*c 68.2) and C-2″ (*δ*c 163.5) (Table 1 and Figure 2). Thus, compound **8** was determined to be 5-hydroxy-8-(2-hydroxypropan-2-yl)-2-(4-methoxyphenyl)-3-(((2S,3R,4R,5R,6S)-3,4,5-trihydroxy-6-methyl-tetrahydro-2H-pyran-2-yl)oxy)-4H-furo[2,3-h]chromen -4-one, named koreanoside G.

### 2.2. Bacterial Neuraminidase Inhibitory Activities

The isolated prenylated flavonoids (**1**–**8**) were tested for enzymatic inhibitory activity against bacterial NA. The enzyme activity was assayed according to a standard literature procedure by following the hydrolysis of 4-methylumbeliferyl-α-d-*N*-acetylneuraminic acid sodium salt hydrate [15]. The inhibitory profiles of the compounds (**1**–**8**) and three mother compounds (luteolin, dihydrokaempferol, and apigenin) are shown in Table 2. All the compounds (**1**–**8**) exhibited a potent bacterial NA inhibition with IC_50_ values of 0.17~106.3 µM.

**Figure 3:**
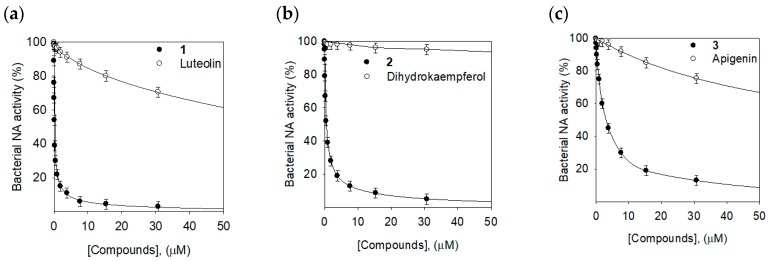
Effectiveness of the prenyl group on the bacterial neuraminidase inhibitory activity. (**a**) compound **1** versus luteolin, (**b**) compound **2** versus dihydrokaempferol, and (**c**) compound **3** versus apigenin.

Three prenylated flavonoids (**1**–**3**) inhibited bacterial NA significantly with IC_50_s of 0.17, 0.68, and 12.6 µM, respectively. They showed much better inhibition than the glycoside compounds (**4**–**6**). In particular, compound **1** (IC_50_ = 0.17 µM), having two prenyl groups on C8 and C5′ of luteolin, was 500 times more effective than the mother skeleton, luteolin (IC_50_ = 85.6 µM), as shown in Figure 3a. Compound **2** (IC_50_ = 0.68 µM) was 700 times more potent in comparison with dihydrokaempferol (IC_50_ = 500.4 µM) (Figure 3b). The prenyl group effectiveness on bacterial NA inhibition was also observed between compound **3** (IC_50_ = 12.6 µM) and apigenin (IC_50_ = 107.5 µM) (Figure 3c). Taken together, the prenyl group on the flavonoids played a critical role in bacterial NA inhibition. The two new compounds (**7** and **8**) also exhibited potent inhibition with IC_50_s of 2.9 µM and 16.7 µM, respectively. Particularly, the structural differences between compounds **7** and **8** was in the presence of the methoxy group or hydroxyl group on C-2″, but the methoxy group (**7**) was 5 times more effective compared with the hydroxyl group (**8**).

**Figure 4:**
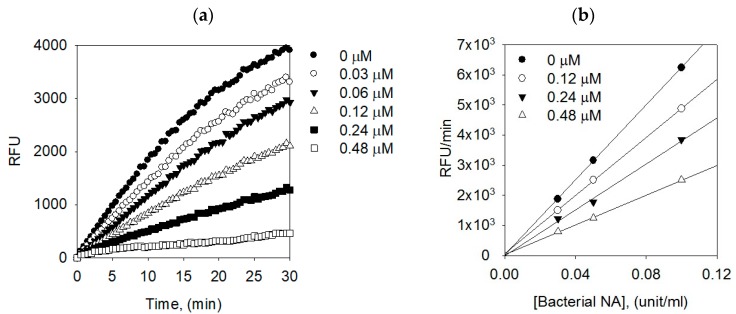
(**a**) Dose-dependent inhibition of bacterial neuraminidase inhibitory effects by compound **1**, and (**b**) Determination of the reversible inhibitory mechanism of compound **1**.

In the kinetic analysis of the inhibitors, all of the compounds manifested the same relationship between enzyme activity and enzyme concentration. All the compounds inhibited the bacterial NA enzyme dose-dependently. The inhibition of bacterial NA by compound **1** (the most effective species) is illustrated in Figure 4a, representatively. On the plots of the initial velocity versus the enzyme concentration in the presence of different concentrations of compound **1**, increasing the inhibitor concentrations resulted in the lowering of the slope of line. All of the straight lines passed through the origin, indicating that this compound was a reversible inhibitor (Figure 4b).

**Figure 5:**
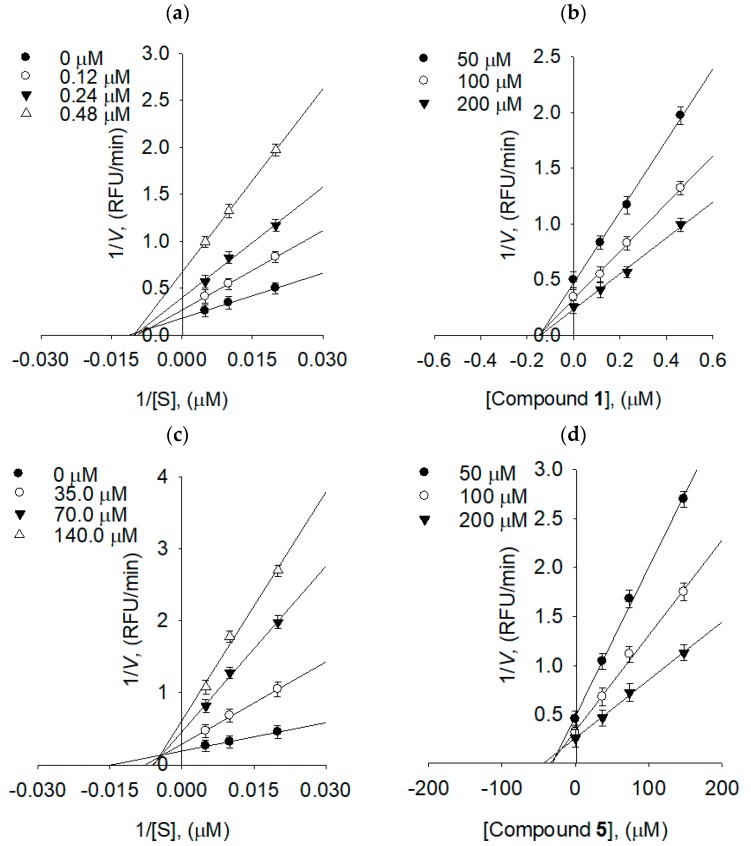
Bacterial neuraminidase kinetics of compounds **1** and **5** using 4-methylumbeliferyl-α-d-*N*-acetylneuraminic acid sodium salt hydrate. (**a**) Lineweaver–Burk plots of compound **1**, (**b**) Dixon plots of compound **1**, (**c**) Lineweaver–Burk plots of compound **5**, and (**d**) Dixon plots of compound **5**.

The enzyme inhibition properties of compounds **1**–**8** were modeled using double-reciprocal plots of Lineweaver–Burk and Dixon plots. As shown in Figure 5a, the analysis of compound **1** exhibited that *V*_max_ decreased without changing *K*_m_ in the presence of the increasing concentration of the inhibitor. As can be seen in the graph (Figure 5), −1/*K*_m_ (the x-intercept) was unaffected by the concentration, whereas 1/*V*_max_ became more positive. This behavior indicates that compound **1** exhibits noncompetitive inhibition characteristics for NA. The *K*_i_ value of compound **1** was calculated as 0.15 µM by Dixon plots (Figure 5b). Similar trends can also be observed in all the compounds bearing sugar moieties on both A and C rings, apart from compound **5**. A similar analysis of compound **5** showed a series of lines, which intercept to the left of the vertical axis and above the horizontal axis, indicating that compound **5** was a mixed-type inhibitor (Figure 5c). The *K*_i_ value was estimated as 36.8 µM by Dixon plots (Figure 5d).

To further confirm noncompetitive and mixed-type behavior, the results were applied to Yang’s method (Table 3) [16]. In this procedure, *K*_m_ and *V*_max_ are plotted against the inhibitor concentration. The new kinetic constant *K*_ik_ can be fitted to Equation (1), while *K*_iv_ can be fitted to Equation (2). From the results of the fit, the *K*_ik_/*K*_iv_ ratio were between 6.68 and 16.04 for compound **1**, which is further consistent with noncompetitive behavior. Compound **5** showed typical mixed-type behavior with 2.02~3.66 of *K*_ik_/*K*_iv_ [16].

### 2.3. Binding Affinities between Bacterial Neuraminidase and Compounds

**Figure 6:**
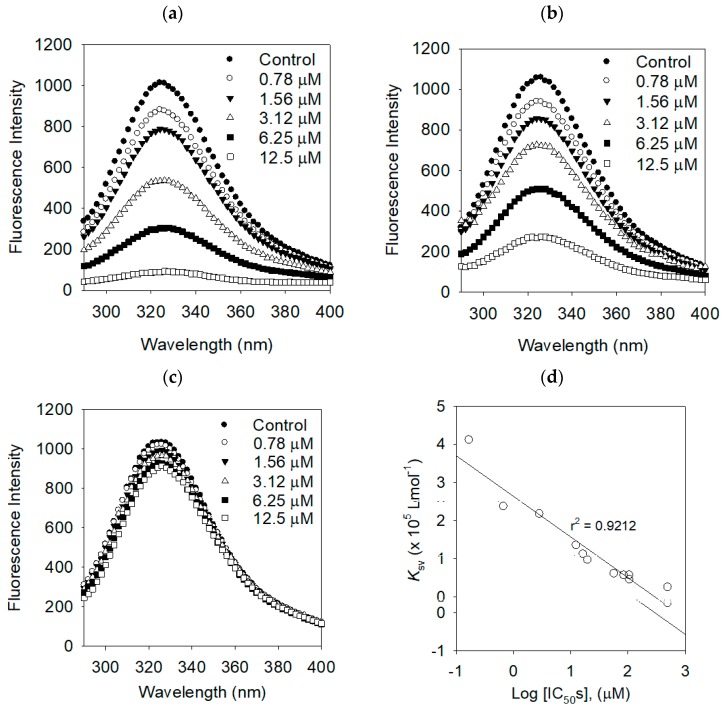
The effects of (**a**) compound **1**, (**b**) compound **2,** (**c**) dihydrokaempferol on the fluorescence emission spectra of bacterial neuraminidase, and (**d**) the correlation between the inhibitory potencies (IC_50_s) and the Stern–Volmer constant (*K*_SV_).

Proteins have intrinsic fluorescence mainly due to tryptophan (Trp), tyrosine (Tyr), and phenylalanine (Phe) residues [17]. The intrinsic fluorescence of the protein often changes as a function of the ligand concentrations. We investigated the enzyme binding affinities of the inhibitors on bacterial NA by a fluorescence quenching (FQ) effect. Bacterial neuraminidase has three fluorescent residues, eight Trp (31, 80, 118, 124, 135, 149, 172, 217, and 264), 22 Tyr (35, 57, 65, 82, 95, 141, 203, 204, 209, 246, 248, 251, 255, 267, 310, 318, 336, 347, 361, 369, 376, and 377), and eight Phe (8, 24, 36, 52, 76, 286, 322, and 352) [18]. There was no significant emission from any of the other components of the assay mixture under the measurement condition (i.e., emission from 300 to 400 nm). The binding affinity (*K*_SV_) was analyzed using the Stern–Volmer Equation (Equation (3)). Figure 6 shows typical Stern–Volmer plots of compounds **1**, **2**, and dihydrokaempferol on bacterial NA. Evidently, the fluorescence intensities were dramatically decreased for the best performing inhibitors **1** (IC_50_ = 0.17 µM), **2** (IC_50_ = 0.68 µM), proportional to the increasing of the concentration. The FQ effect of the lowest active compound (dihydrokaempferol, IC_50_ = 500.4 µM) was insignificant. The FQ effects of the remaining compounds are displayed in Table 4 and the Supplementary Materials. The binding affinities of the Stern–Volmer constants (*K*_SV_) of inhibitors could be ranked in the following order 1 > 2 > 3 > 4, which is in agreement with the order of their inhibition potencies (IC_50_s) as shown in Figure 6d. The binding constant (*K*_A_) and the number of binding sites (*n*) were calculated by Equation (4). The binding constants (*K*_A_) were also correlated with inhibitory potencies (IC_50_s) as shown in Table 4.

## 3. Materials and Methods

### 3.1. Chemicals and Materials

The organic solvents used for isolation were of first grade. Water, methanol, acetonitrile, and acetic acid of analytical grade for HPLC and MPLC were purchased from Fisher (Fisher Scientific, Hampton, NH, USA). Open column chromatography was carried out using silica gel (230–400 mesh, Merck, Kenilworkth, NJ, USA), C-18S (12 nm, S-20 µm, YMC, Kyoto, Japan), and Sephadex LH-20 (GE Healthcare Life Sciences, Chicago, IL, USA). Triart C18 (S-5 µm, 12 nm and S-10 µm, 12 nm, YMC, Kyoto, Japan) was used for recycle HPLC and MPLC. The enzyme assay was carried out with neuraminidase from *Clostridium perfringens* (EC 3.2.1.18) (Sigma Aldrich Co., St. Louis, MO, USA). *E. koreanum* Nakai leaves (1.8 kg) permitted by Korea Food and Drug Administration (KFDA) were purchased from a local market.

### 3.2. Instruments

The UV spectra were measured in Spectra Max M3 Multi-Mode Microplate Reader (Molecular Devise, Sunnyvale, CA, USA). ^1^H and ^13^C-NMR, as well as 2D NMR data, were obtained on a Bruker AM 500 spectrometer (Bruker, Karlsruhe, Germany). High-resolution fast atom bombardment mass (HRFABMS) spectra were obtained on a JEOL JMS-700 instrument (JEOL Ltd., Akishima, Japan). MPLC was conducted on a Forte/R 100 (YMC Co., Ltd., Kyoto, Japan) and recycle HPLC was conducted on a LC-9130G NEXT (JAI Co., Ltd., Tokyo, Japan).

### 3.3. Extraction and Isolation

The dried leaves of *E. koreanum* Nakai (0.5 kg) were extracted using methanol (10 l) at room temperature for 1 week to obtain a crude extract (48 g). The crude extract was suspended in water and successively partitioned into ethyl acetate to afford a dark residue (18 g). The ethyl acetate fraction (15 g) was subjected to column chromatography on silica gel (8 × 40 cm, 500 g) and eluted with a gradient flow of *n*-hexane/ethyl acetate (20:1 to 1:2, *v*/*v*) to give 20 fractions (A1–A20, each 200 mL). The fractions A8–15 (6.8 g) were fractionated via Forte-R MPLC (250 × 20.0 mm, C18-S, S-10 µm, 12 nm, YMC) eluting with a gradual increase in MeOH (0–100%) to afford 50 subfractions (B1–B50, 20 mL each). The above MPLC process was repeated with 0.5 g each time. Subfractions B12–16 (1.8 g) enriched with compounds **4**–**8** were further chromatographed over LC-9130G NEXT recycle HPLC (250 × 20.0 mm, C18-S, S-5 µm, 12 nm, YMC, Kyoto, Japan), eluted with a gradual increase in MeOH (0–100%). This recycle HPLC was repeated to afford compounds **4** (22 mg), **5** (18 mg), and **6** (21 mg). The mixture enriched with compounds **7** and **8** was purified by Sephadex LH-20 with eluting MeOH to obtain the compounds **7** (10 mg) and **8** (12 mg). Similarly, subfractions B17–21 (1.1 g), enriched with compounds **1**–**3**, were used in the recycle HPLC to yield compounds **1** (11 mg), **2** (16 mg), and **3** (8 mg). All the isolated compounds were identified on the basis of spectroscopic data and comparisons with previous studies (Supplementary Materials) [15,19,20].

#### 3.3.1. Epimedokoreanin B (Compound **1**)

EIMS *m*/*z* 422 [M]^+^; HREIMS m/z 422.1721 (Calcd for C_25_H_26_O_6_, 422.1729); pale yellow powder; ^1^H-NMR (500 MHz, MeOD); δ_H_ 1.57 (3H, d, *J* = 0.8 Hz, H-5″), 1.64 (3H, s, H-5‴), 1.66 (3H, d, *J* = 0.9 Hz, H-4″), 1.69 (3H, s, H-4‴), 3.25 (2H, d, *J* = 7.3 Hz, H-1″), 3.40 (2H, d, *J* = 7.1 Hz, H-1‴), 5.18 (1H, m, H-2″), 5.24 (1H, dddd, *J* = 7.4, 6.0, 2.8, 1.4 Hz, H-2‴), 6.13 (1H, s, H-6), 6.36 (1H, s, H-3), 7.13 (1H, d, *J* = 2.1 Hz, H-2′), 7.21 (1H, d, *J* = 2.2 Hz, H-6′).

#### 3.3.2. 8-(γ,γ-Dimethyl allyl)-5,7,4′-trihydroxydihydroflavonol (Compound **2**)

EIMS *m*/*z* 356 [M]^+^; HREIMS *m*/*z* 356.1262 (Calcd for C_20_H_20_O_6_, 356.1260); yellow powder; ^1^H-NMR (500 MHz, MeOH); δ_H_ 1.40 (3H, s, H-5″), 1.50 (3H, s, H-4″), 3.03 (2H, s, H-1″), 4.39 (1H, s, H-3), 4.82 (1H, s, H-2), 5.01 (1H, s, H-2″), 6.74 (2H, d, *J* = 8.6 Hz, H-3′ and H-5′), 7.26 (2H, d, *J* = 8.5 Hz, H-2′ and H-6′), 8.87 (1H, s, H-6).

#### 3.3.3. 5,7,4′-Trihydroxy-8,3′-diprenylflavone (Compound **3**)

EIMS *m*/*z* 406 [M]^+^; HREIMS *m*/*z* 406.1784 (Calcd for C_25_H_26_O_5_, 406.1780); white powder; ^1^H-NMR (500 MHz, MeOH); δ_H_ 1.56 (3H, s, H-5‴), 1.62 (3H, s, H-5″), 1.66 (3H, s, H-4‴), 1.68 (3H, s, H-4″), 3.22 (2H, s, H-1″), 3.34 (2H, d, *J* = 6.9 Hz, H-1‴), 5.13 (1H, t, *J* = 6.9 Hz, H-2‴), 5.22 (1H, t, *J* = 7.4 Hz, H-2″), 6.10 (1H, s, H-6), 6.34 (1H, s, H-3), 6.74 (1H, d, *J* = 8.3 Hz, H-3′), 7.48 (1H, d, *J* = 6.5 Hz, H-2′), 7.55 (1H, d, *J* = 2.1 Hz, H-6′).

#### 3.3.4. Icariside II (Compound **4**)

FABMS *m*/*z* 515 [M]^+^; HRFABMS m/z 515.1893 (Calcd for C_27_H_30_O_10_, 515.1839); pale yellow powder; ^1^H-NMR (500 MHz, MeOH); δ_H_ 0.92 (3H, d, *J* = 6.0 Hz, H-6‴), 1.68 (3H, s, H-5″), 1.73 (3H, s, H-4″), 3.29 (1H, dd, *J* = 9.6, 6.0 Hz, H-4‴), 3.34 (2H, dd, *J* = 20.5, 6.8 Hz, H-1″), 3.36 (1H, m, H-5‴), 3.73 (1H, dd, H-3‴), 3.91 (3H, s, H-7′), 4.24 (1H, d, *J* = 1.4 Hz, H-2‴), 5.21 (1H, t, *J* = 6.9 Hz, H-2″), 5.46 (1H, ddd, *J* = 35.2, 14.8, 7.0 Hz, H-1‴), 6.28 (1H, s, H-6), 7.10 (2H, d, *J* = 8.8 Hz, H-3′ and H-5′), 7.89 (2H, d, *J* = 8.8 Hz, H-2′ and H-6′).

#### 3.3.5. Icariin (Compound **5**)

FABMS *m*/*z* 677 [M]^+^; HRFABMS *m*/*z* 677.2460 (Calcd for C_33_H_40_O_15_, 677.2367); pale yellow powder; ^1^H-NMR (500 MHz, DMSO); δ_H_ 0.79 (3H, d, *J* = 6.03, H-6″″), 1.60 (3H, s, H-5″), 1.69 (3H, s, H-4″), 3.20–3.02 (3H, m, H-4‴), 3.20–3.02 (3H, m, H-4″″), 3.20–3.02 (3H, m, H-5″″), 3.31*–*3.27 (2H, m, H-2‴), 3.31–3.27 (2H, m, H-3‴), 3.43*–*3.40 (2H, m, H-5‴), 3.48 (1H, dd, *J* = 5.81, 11.73 Hz, H-3″″), 3.57(1H, dd, *J* = 7.46, 14.56 Hz, H-1″), 3.71 (1H, dd, *J* = 10.2, 5.3 Hz, H-6‴), 3.86 (3H, s, H-7′), 4.00(1H, t, *J* = 4.44 Hz, H-2″″), 4.99 (1H, d, *J* = 4.41Hz, H-1‴), 5.28 (1H, d, *J* = 1.45 Hz, H-1″″), 6.63 (1H, s, H-6), 7.13 (2H, d, *J* = 9.0 Hz, H-3′ and H-5′), 7.89 (2H, d, *J* = 8.9 Hz, H-2′ and H-6′).

#### 3.3.6. Sagittatoside B (Compound **6**)

FABMS *m/z* 647 [M]^+^; HRFABMS *m*/*z* 647.2369 (Calcd for C_32_H_38_O_14_, 647.2262); pale yellow powder; ^1^H-NMR (500 MHz, MeOD); δ_H_ 0.97 (2H, d, *J* = 6.2 Hz, H-6‴), 1.66 (3H, s, H-5″), 1.71 (3H, s, H-4″), 3.06 (1H, t, *J* = 11.0 Hz, H-5″″b), 3.22*–*3.03 (1H, m, H-3‴), 3.22*–*3.17 (1H, m, H-3″″), 3.33*–*3.26 (2H, m, H-2‴), 3.33*–*3.26 (2H, m, H-2″″), 3.42*–*3.37 (2H, m, H-4″″), 3.44 (1H, dd, *J* = 8.3 Hz, H-1″), 3.61 (1H, dd, *J* = 9.6, 6.2 Hz, H-5‴), 3.67 (1H, dd, *J* = 5.4, 11.5 Hz, H-5″″a), 3.82 (1H, dd, *J* = 3.4, 9.8 Hz, H-4‴), 3.86 (3H, s, H-7′), 4.30 (1H, d, *J* = 7.63Hz, H-1″″), 5.19 (1H, t, *J* = 6.87 Hz, H-2″), 5.46 (1H, s, H-1‴), 6.26 (1H, s, H-6), 7.11 (2H, d, *J* = 8.9 Hz, H-3′ and H-5′), 7.87 (2H, d, *J* = 8.9 Hz, H-2′ and H-6′).

#### 3.3.7. Koreanoside F (Compound **7**)

FABMS *m*/*z* 543 [M]^+^; HRFABMS *m*/*z* 543.1906 (Calcd for C_28_H_31_O_11_, 543.1788); yellow powder; for ^1^H and ^13^C-NMR data, see Table 1.

#### 3.3.8. Koreanoside G (Compound **8**)

FABMS *m*/*z* 529 [M]^+^; HRFABMS *m*/*z* 529.1682 (Calcd for C_27_H_28_O_11_, 529.1632); yellow powder; for ^1^H and ^13^C-NMR data, see Table 1.

### 3.4. Bacterial Neuraminidase Inhibitory Activity Assay

Neuraminidase from *Clostridium perfringens* (*C. welchii*) (EC 3.2.1.18) was evaluated as described previously with slight modification [21]. 4-Methylumbelliferyl-*N*-acetyl-α-d-neuraminic acid sodium salt hydrate was used as the substrate. The fluorescence was measured using a UV–Vis spectrophotometer (Spectra Max M3, Molecular Devise, Sunnyvale, CA, USA) with a 96-well black immuno-microplate (SPL life science, Pocheon, Korea) at 37 °C with an emission wavelength of 450 nm and an excitation wavelength of 365 nm. All the samples were dissolved in DMSO at 10 mM and diluted to the required concentration. First, 20 µl of 1 mM of an aqueous solution of the substrate (*K*_m_ = 100 µM) was mixed with 160 µl of 50 mM sodium acetate buffer (pH 5.0). Then, 10 µl of the inhibitors and 10 µl of neuraminidase (0.2 units/mL) were added respectively to the mixture. Each assay was conducted as 3 separate replicates. The inhibitor concentration, leading to a 50% activity loss (IC_50_), was obtained by the following equation: Activity (%) = 100 [1 + ([I]/IC_50_)].

### 3.5. Enzyme Kinetics and Progress Linear Determinations

To determine the enzyme inhibition kinetics, an experiment was performed having different substrate and inhibitor concentration ranges. To find out each curve parameter, a nonlinear regression program was used for data analysis using Sigma Plot. Similarly, *K*_m_ and *V*_max_ were derived from the Lineweaver–Burk plot. Additionally, the *K*_i_ value was calculated from Dixon plots. The *K*_ik_ and *K*_iv_ rate constants were calculated according to Equations (1) and (2) proposed by Yang et al. [16].

*K*_m_ = *K*_m0_ × (1 + [I]/*K*_ik_) (1)

*V*_m_ = *V*_m0_ × (1 + [I]/*K*_iv_) (2)

### 3.6. Binding Affinity between Bacterial Neuraminidase and Compounds

180 µl of 50 mM sodium acetate buffer (pH 5.0) with 10 µl of 0.5 unit/mL neuraminidase from *Clostridium perfringens* were accurately added into the 96-well black immuno-plates; then, different 10 µl concentrations (15.6~250 µM) of inhibitor were added. The spectra for the fluorescent emissions were recorded from 300 to 400 nm with emission slits adjusted to 2.0 nm, and the excitation was 260 nm using a spectrophotometer (Spectra Max M3). All the experiments were performed in triplicate. Fluorescence quenching is described by the Stern–Volmer equation.

*F*_0_ − *F* = 1 + *K*_SV_[Q] (3)

*F*_0_ and *F* are the fluorescence intensities in the absence and presence of quencher (Q). *K*_SV_ is the Stern–Volmer quenching constant [lM^−1^]. For static quenching, the relationship between the change in the fluorescence intensity and the concentration of quencher for the set of reaction can be described by the following equation.

log[(*F*_0_ − *F*)/*F*] = log*K*_A_ + *n*log[Q]_f_ (4)

Q_f_ is the concentration of free inhibitors; *n* is the number of binding sites; and *K*_A_ is the binding constant. From the plots of linear Equation (4) obtained by log [(*F*_0_ − *F*)/*F*] versus log [Q]_f_, one can calculate the values of *K*_A_ and *n.* The value of *n* approximates to one, indicating that only a single binding site exists in bacterial neuraminidase for inhibitors [22].

### 3.7. Statistical Analysis

All the experiments were conducted in triplicate. The results were subjected to variance analysis using Sigma Plot (version 10.0, Systat Software, Inc., San Jose, CA, USA). Differences were considered significant at *p* < 0.05.

## 4. Conclusions

In conclusion, we have undertaken a thorough investigation of bacterial neuraminidase inhibition by *E. koreanum* Nakai, an important medicinal plant. The principal components were identified as prenylated flavonoids, including two new ones, named koreanoside F and koreanoside G. They showed mainly noncompetitive behavior that was demonstrated with the kinetic parameters *V*_max_, *K*_m_, *K*_ik_, and *K*_iv_. The binding affinities (*K*_SV_) of the inhibitors were measured by a fluorescence quenching effect. In particular, the prenyl group on the flavonoids played a critical role in bacterial NA inhibition. The most active compound **1** (IC_50_ = 0.17 µM) was 500 times more effective than its mother skeleton (IC_50_ = 85.6 µM). We are hopeful that prenylated flavonoids can be of use as a lead structure for neuraminidase inhibitors.

## Figures and Tables

**Table 1:** ^1^H-NMR and ^13^C-NMR data of compounds **7** and **8** (500 MHz, MeOD).

Position	7		8	
	*δ*_H_, mult, (*J*, Hz)	*δ*c	*δ*_H_, mult, (*J*, Hz)	*δ*c
2	-	157.7	-	157.5
3	-	135.9	-	135.8
4	-	179.1	-	179.2
5	-	159.5	-	157.8
6	6.78, s	94.2	6.90, s	94.2
7	-	148.9	-	148.9
8	-	108.9	-	109.2
9	-	158.2	-	158.9
10	-	107.4	-	107.3
1′	-	122.2	-	122.2
2′,6′	7.88, d, (*J* = 8.7 Hz)	130.6	7.98, d, (*J* = 8.8 Hz)	130.5
3′,5′	7.04, d, (*J* = 8.7 Hz)	113.9	7.15, d, (*J* = 8.8 Hz)	113.9
4′	-	162.3	-	162.3
1″	6.93, s	100.6	6.91, s	96.9
2″	-	159.1	-	163.5
3″	-	73.3	-	68.2
4″	1.53, s	24.4	1.66, s	27.5
5″	1.53, s	24.1	1.66, s	27.5
1‴	5.38, s	102.2	5.48, s	102.2
2‴	3.19, overlap	70.5	3.30, overlap	70.5
3‴	4.16, m	70.7	4.27, d, (*J* = 1.7 Hz)	70.7
4‴	3.63, m	70.8	3.75, m	70.8
5‴	3.25, overlap	71.7	3.37, overlap	71.7
6‴	0.82, d, (*J* = 5.8 Hz)	16.3	0.93, d, (*J* = 5.90 Hz)	16.3
3″-OCH_3_	3.03, s	49.9	-	-
4′-OCH_3_	3.81, s	54.6	3.93, s	54.6

**Table 2:** Inhibitory effects of the compounds (**1**–**8**) on bacterial neuraminidase activity.

Compounds	Bacterial Neuraminidase	
	IC_50_^1^ (µM)	Kinetic Mode (*K*_i_^2^, µM)
**1**	0.17 ± 0.02	Noncompetitive (0.15 ± 0.01)
**2**	0.68 ± 0.03	Noncompetitive (0.73 ± 0.03)
**3**	12.6 ± 0.2	Noncompetitive (12.2 ± 0.3)
**4**	20.0 ± 0.5	Noncompetitive (21.1 ± 0.8)
**5**	57.8 ± 1.1	Mixed type I (36.8 ± 0.7)
**6**	106.3 ± 3.2	Noncompetitive (103.2 ± 1.4)
**7**	2.90 ± 0.7	Noncompetitive (2.73 ± 0.6)
**8**	16.7 ± 0.9	Noncompetitive (16.4 ± 0.9)
Luteolin	85.6 ± 2.1	NT ^3^
Dihydrokaempferol	500.4 ± 3.8	NT
Apigenin	107.5 ± 1.8	NT
Quercetin ^4^	20.2 ± 0.8	NT

^1^ All the compounds were examined in a set of experiments repeated three times; IC_50_ values of compounds represent the concentration that caused 50% enzyme activity loss. ^2^ Values of inhibition constant. ^3^ NT means not tested. ^4^ Quercetin was used as a positive control.

**Table 3:** Effect of different concentrations of compounds **1** and **5** on *V*_max_, *K*_m_, and the *K*_ik_/*K*_iv_ ratio using bacterial neuraminidase.

Compounds	[I], (µM)	*V*_max_^1^	*K*_m_^2^	*K*_ik_/*K*_iv_^3^
**1**	0	5.531	107.4564	-
-	0.12	2.738	105.2853	16.0415
-	0.24	2.513	98.6856	6.6846
-	0.48	1.494	97.3510	7.7610
**5**	0	5.1813	120.1982	-
-	35.0	3.4578	131.0961	3.6688
-	70.0	2.1692	150.0805	2.3384
-	140.0	1.6200	161.0846	2.0207

^1^*V*_max_ is the maximal velocity of the enzyme assay. ^2^ *K*_m_ is the Michaelis–Menten constant. ^3^ The *K*_ik_/*K*_iv_ ratio is a rate constant according to Yang’s method [16].

**Table 4:** Fluorescence quenching effects of the isolated compounds (**1**–**8**) and their parent compounds (luteolin, dihydrokaempferol, and apigenin) on the bacterial neuraminidase.

Compounds	*K*_SV_ (× 10^5^ L·mol^−1^)	R^2^	*K*_A_ (× 10^6^ L·mol^−1^)	*n*	R^2^
**1**	4.1104	0.9886	0.9666	1.6920	0.9917
**2**	2.3636	0.9843	0.7923	1.1974	0.9933
**3**	1.3442	0.9997	0.7604	1.1206	0.9915
**4**	0.9569	0.9989	0.7335	1.0917	0.9998
**5**	0.6040	0.9920	0.5844	0.8147	0.9955
**6**	0.5608	0.9875	0.5057	0.7006	0.9998
**7**	2.1656	0.9895	0.7851	1.1733	0.9955
**8**	1.1120	0.9904	0.7416	1.0868	0.9986
Luteolin	0.5614	0.9986	0.5578	0.7662	0.9998
Dihydrokaempferol	0.2479	0.9959	0.2998	0.5758	0.9986
Apigenin	0.4481	0.9926	0.5142	0.4481	0.9993

## Supplementary Materials

The following are available online, Figures S1–S32: 1D, 2D-NMR of isolated compounds (**1**–**8**), Figure S33: Enzyme kinetic data of isolated compounds, Figure S34: Fluorescence quenching effect of isolated compounds.
