# Supplementary material for: Effectiveness of Prenyl Group on Flavonoids from Epimedium koreanum Nakai on Bacterial Neuraminidase Inhibition
Source: Molecules. 2019 Jan 16;24(2):317. doi: 10.3390/molecules24020317 (PMC6359343; doi:10.3390/molecules24020317)
Supplement: Supplementary file 1 [file molecules-24-00317-s001.pdf]

Supplementary materials

Effectiveness of prenyl group on flavonoids from *Epimedium koreanum* to bacterial neuraminidase inhibition

Hong Min Choi, Jeong Yoon Kim, Zuo Peng Li, Janar Jenis, Yeong Jun Ban, Aizhamal Baiseitova, Ki Hun Park*

Division of Applied Life Science (BK21 plus), IALS, Gyeongsang National University, Jinju 52828, Republic of Korea

- **Figure S1-32**: 1D, 2D-NMR of isolated compounds **1-8**
- **Figure S33-34**: Enzyme kinetic data of isolated compounds (**2-4** and **6-8**)
- **Figure S35**: Fluorescence quenching effect of compounds (**3-8**, luteolin, and apigenin)

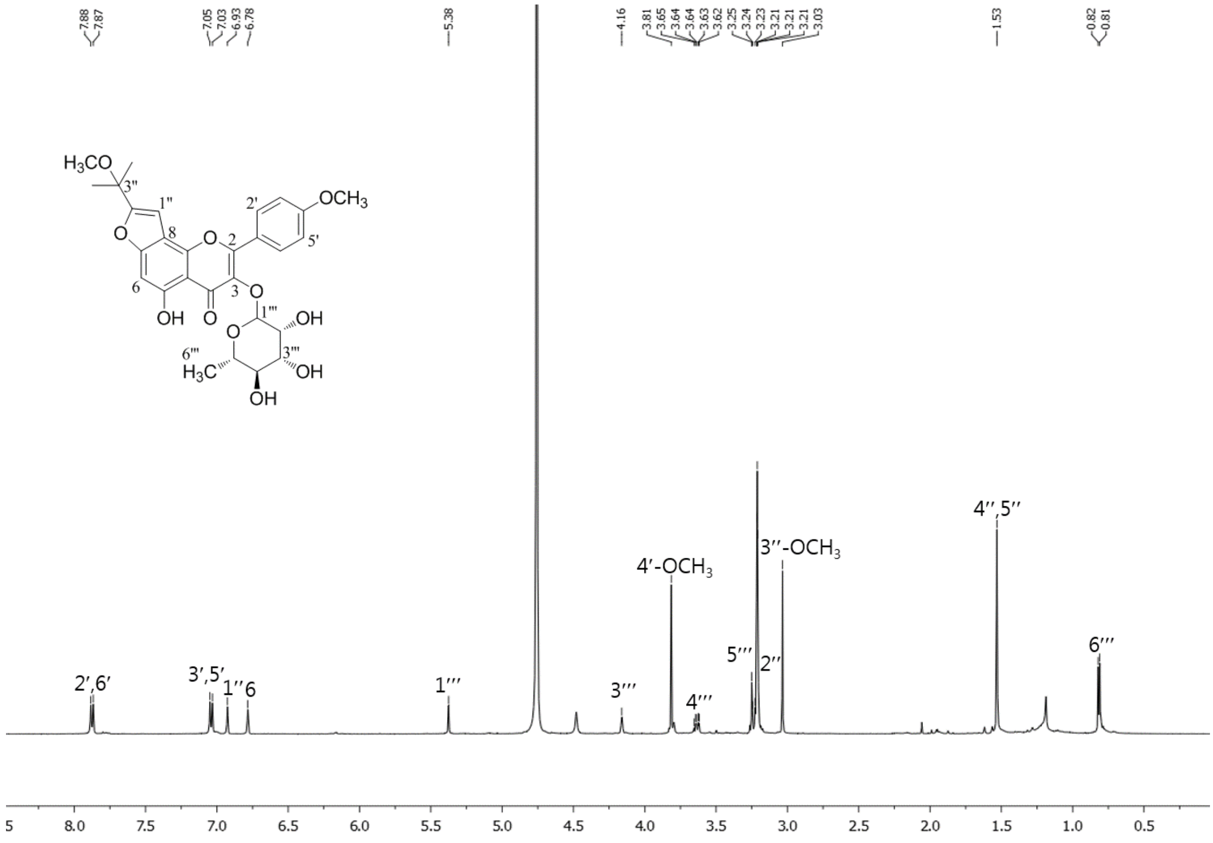

Figure 1. ^{1}H NMR spectrum of compound **7** (500 MHz, MeOD).

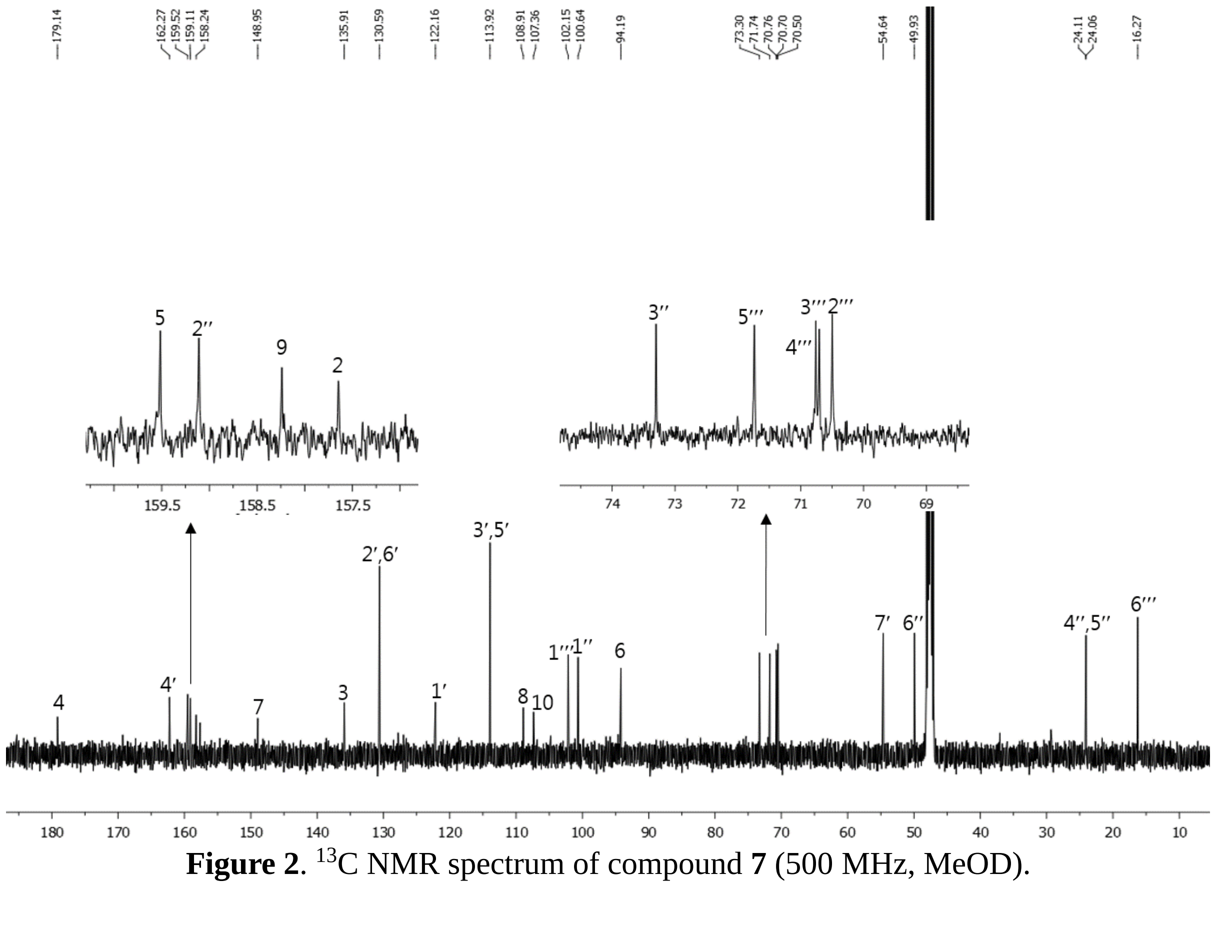

Figure 2. ^{13}C NMR spectrum of compound **7** (500 MHz, MeOD).

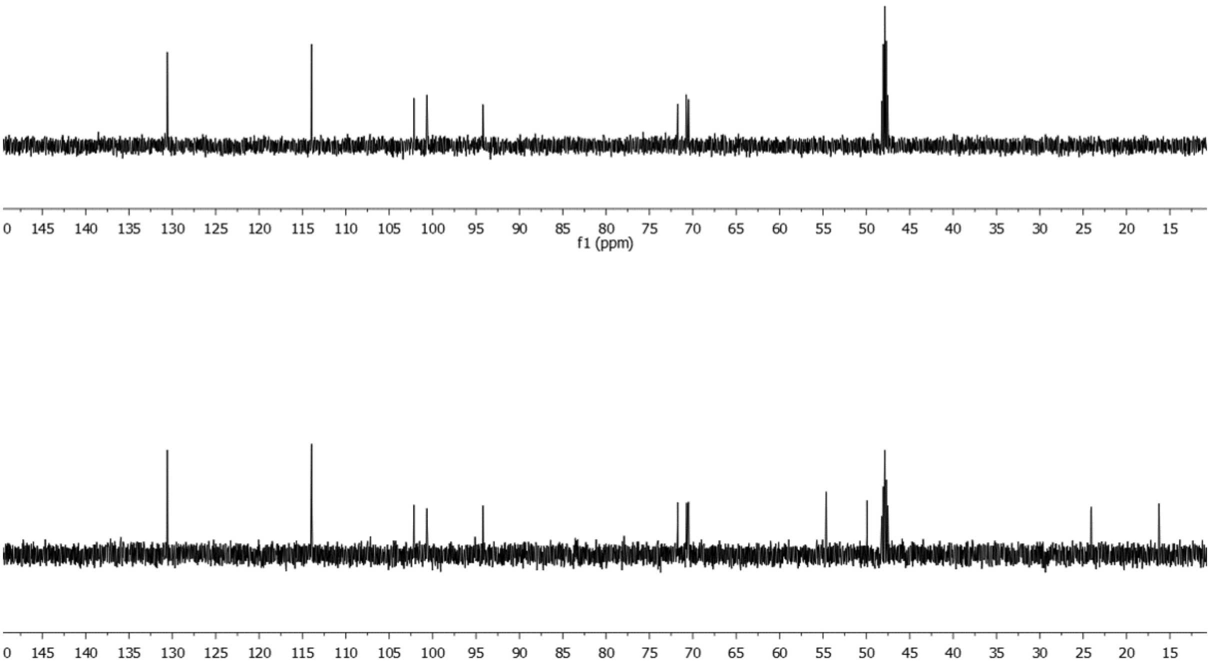

Figure 3. DEPT-90 and -135 spectrum of compound **7** (500 MHz, MeOD).

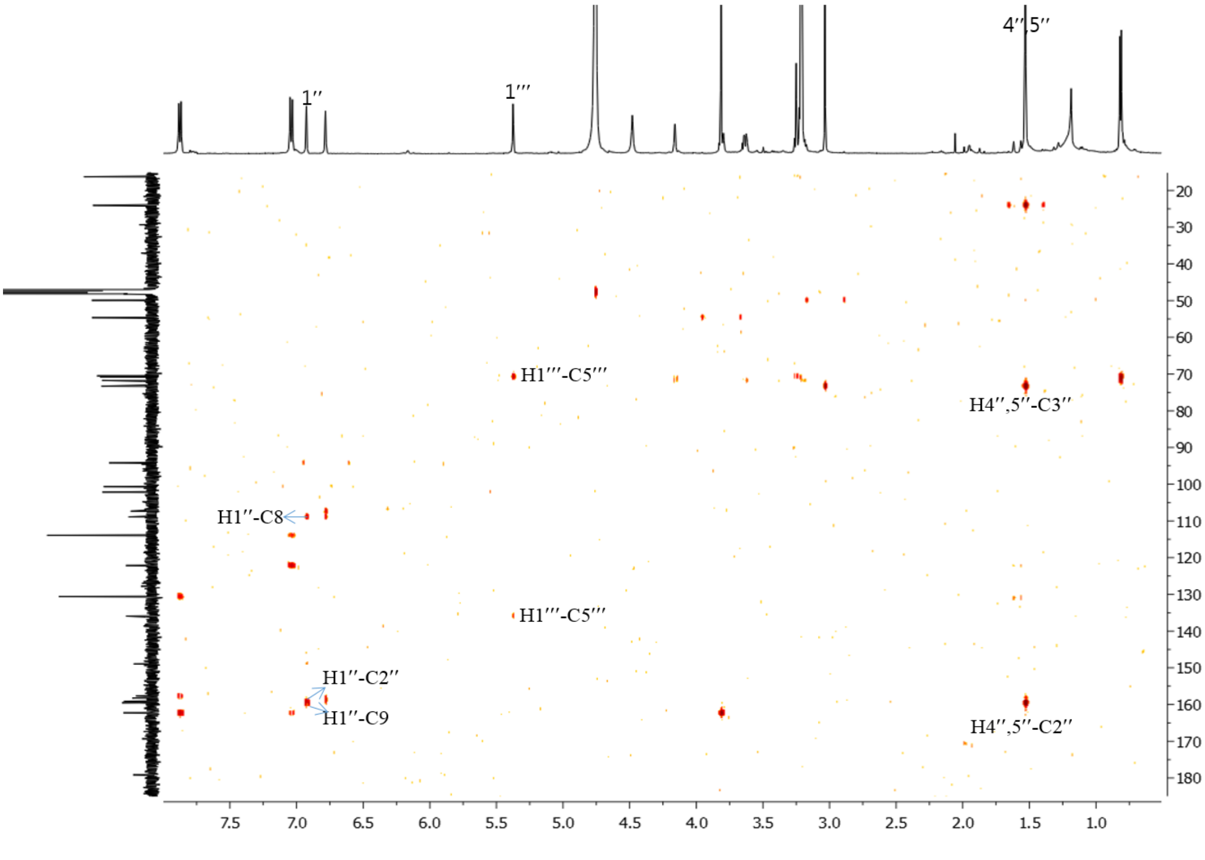

Figure 4. HMBC spectrum of compound **7** (500 MHz, MeOD).

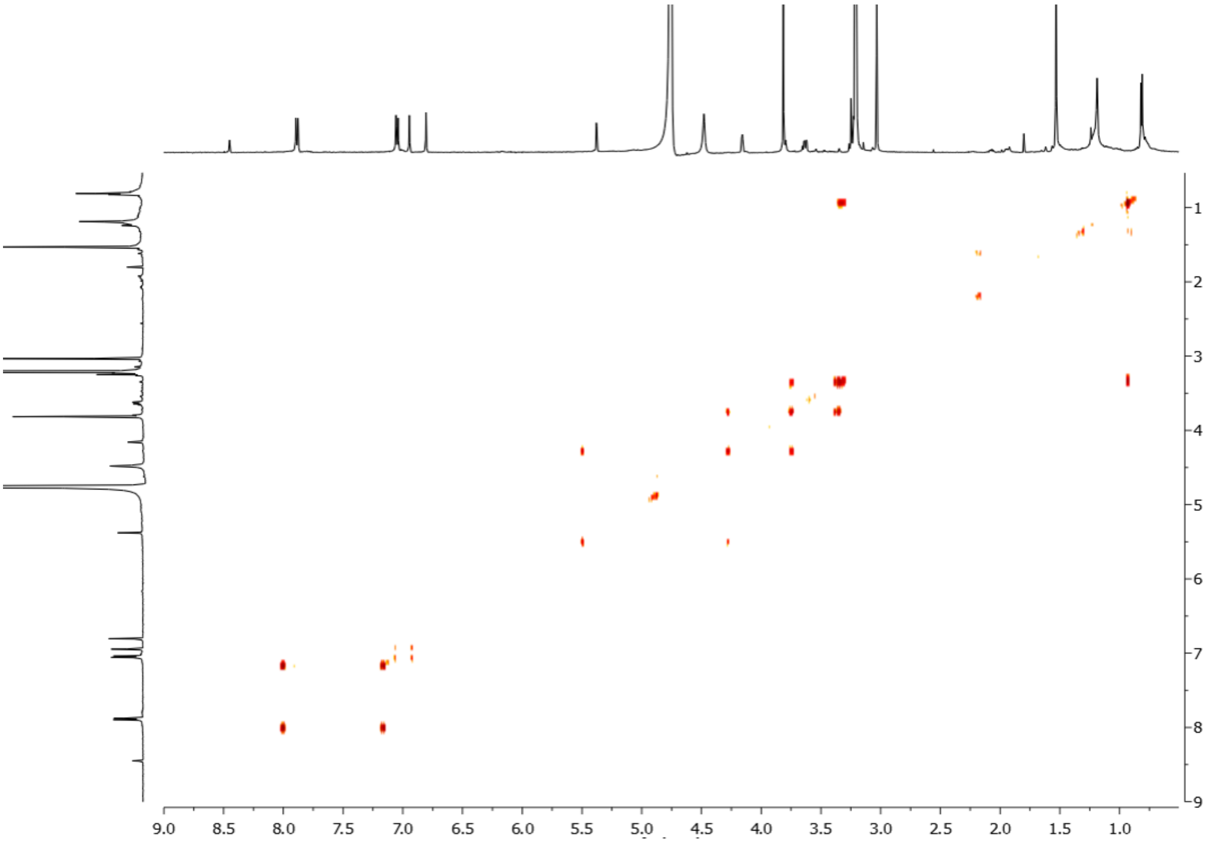

Figure 5. COSY spectrum of compound **7** (500 MHz, MeOD).

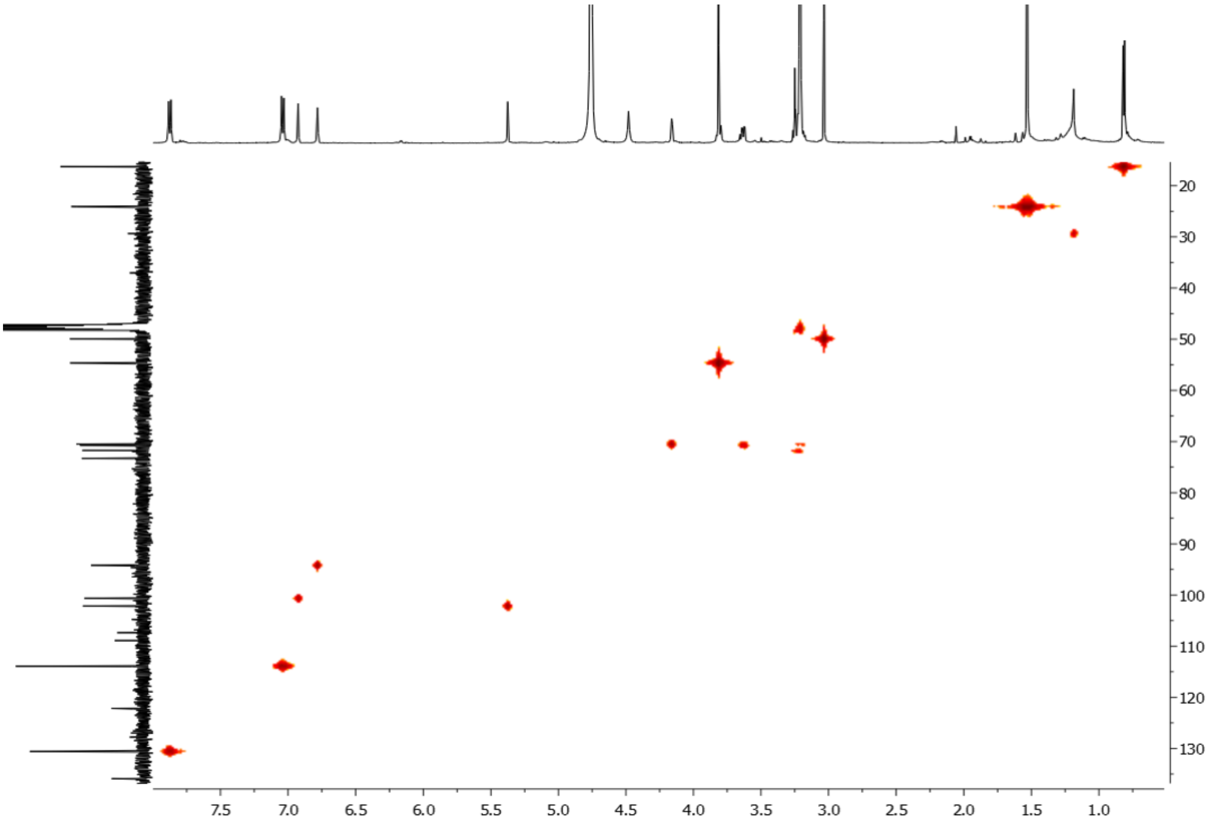

Figure 6. HMQC spectrum of compound **7** (500 MHz, MeOD).

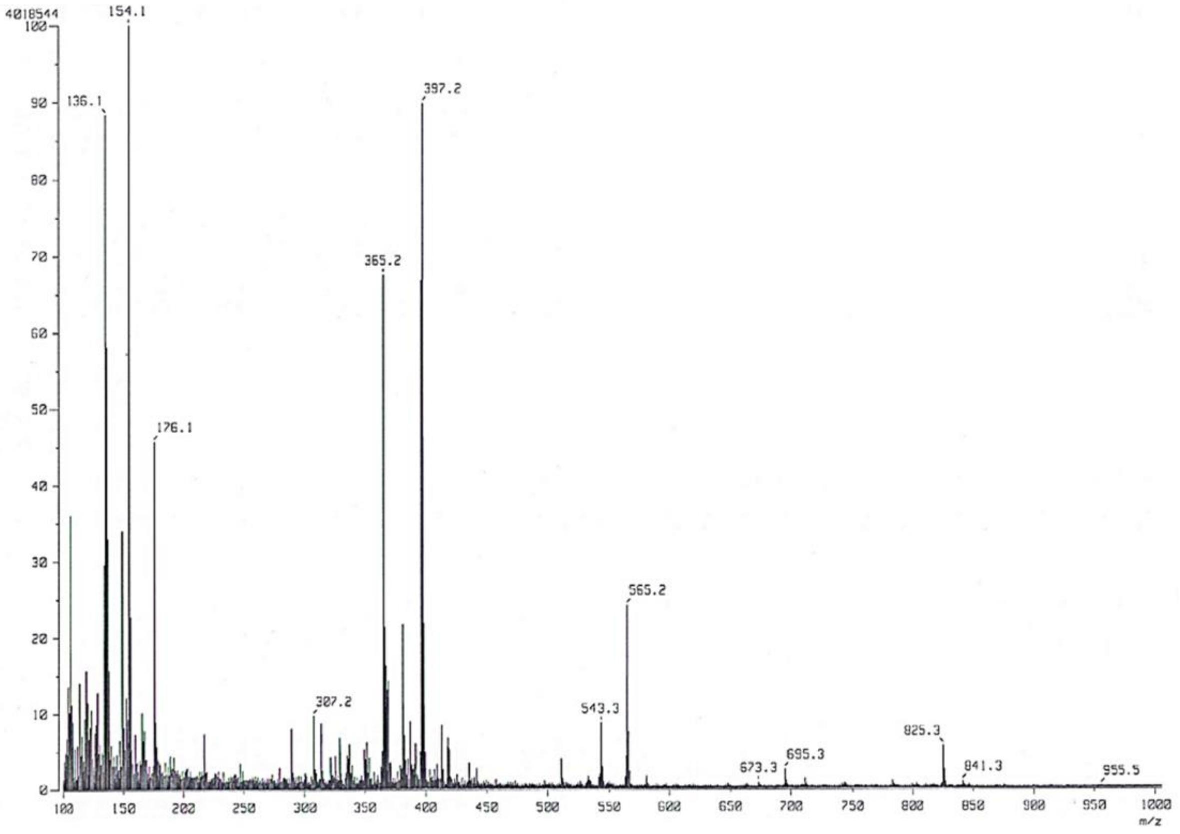

[Elemental Composition]

Data : HM-7-HR1

Date : 05-Apr-2018 16:38

Sample: -

Note : -

Inlet : Direct

Ion Mode : FAB+

RT : 1.00 min

Scan#: 13

Elements : C 100/1, H 100/1, O 20/1

Mass Tolerance : 100ppm, 10mmu if m/z > 100

Unsaturation (U.S.) : 0.0 - 20.0

Observed m/z	Int%	Err[ppm / mmu]	U.S.	Composition
543.1906	11.9	+7.2 / +3.9	13.5	C 28 H 31 O 11
		-3.6 / -2.0	4.5	C 21 H 35 O 16

Figure 7. FABMS spectra and HRFABMS data of compound **7**.

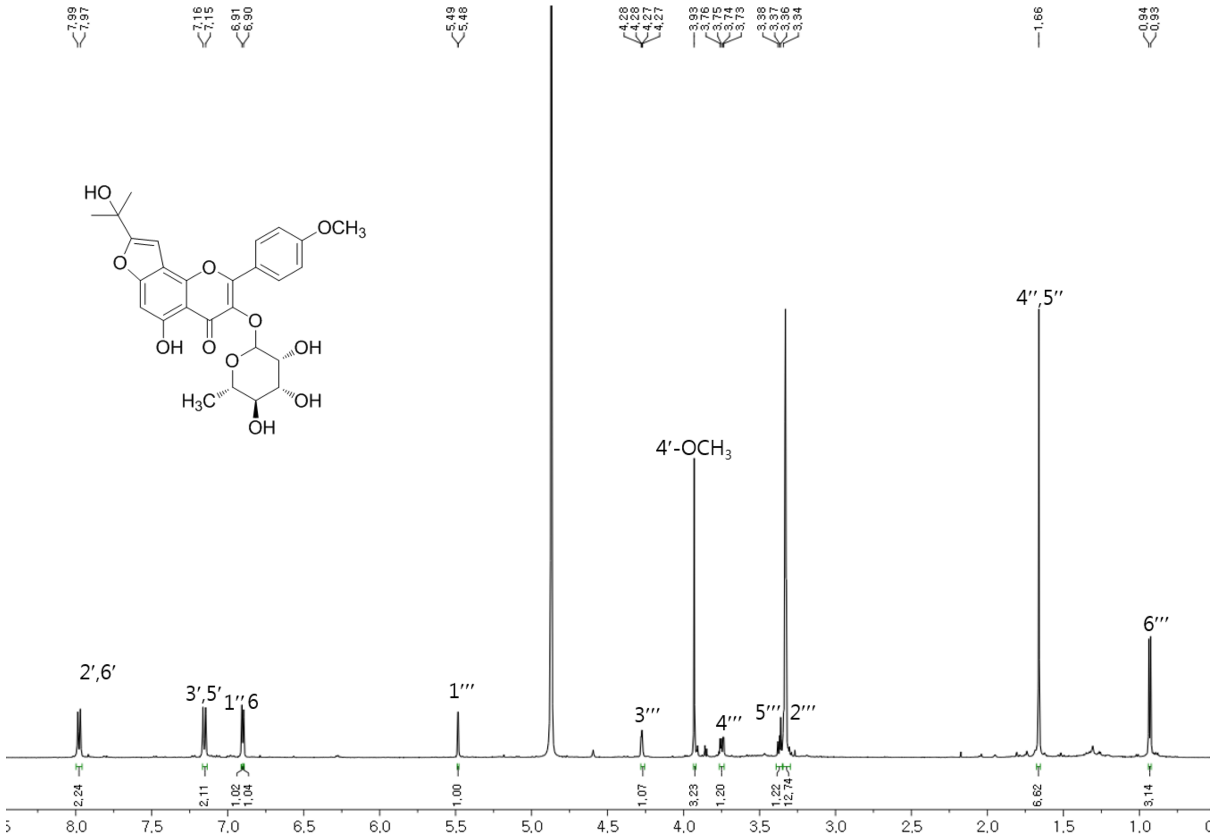

Figure 8. ^{1}H NMR spectrum of compound **8** (500 MHz, MeOD).

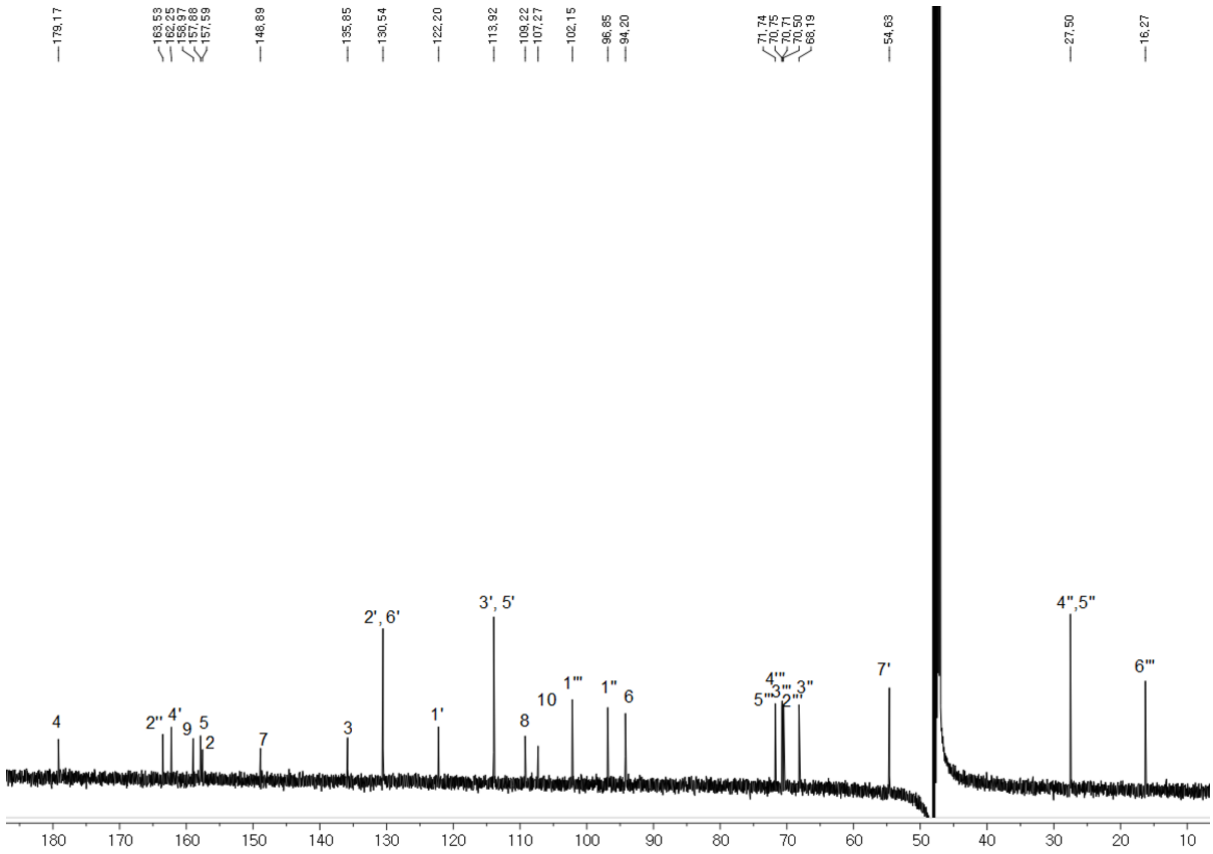

Figure 9. ^{13}C NMR spectrum of compound **8** (125 MHz, MeOD).

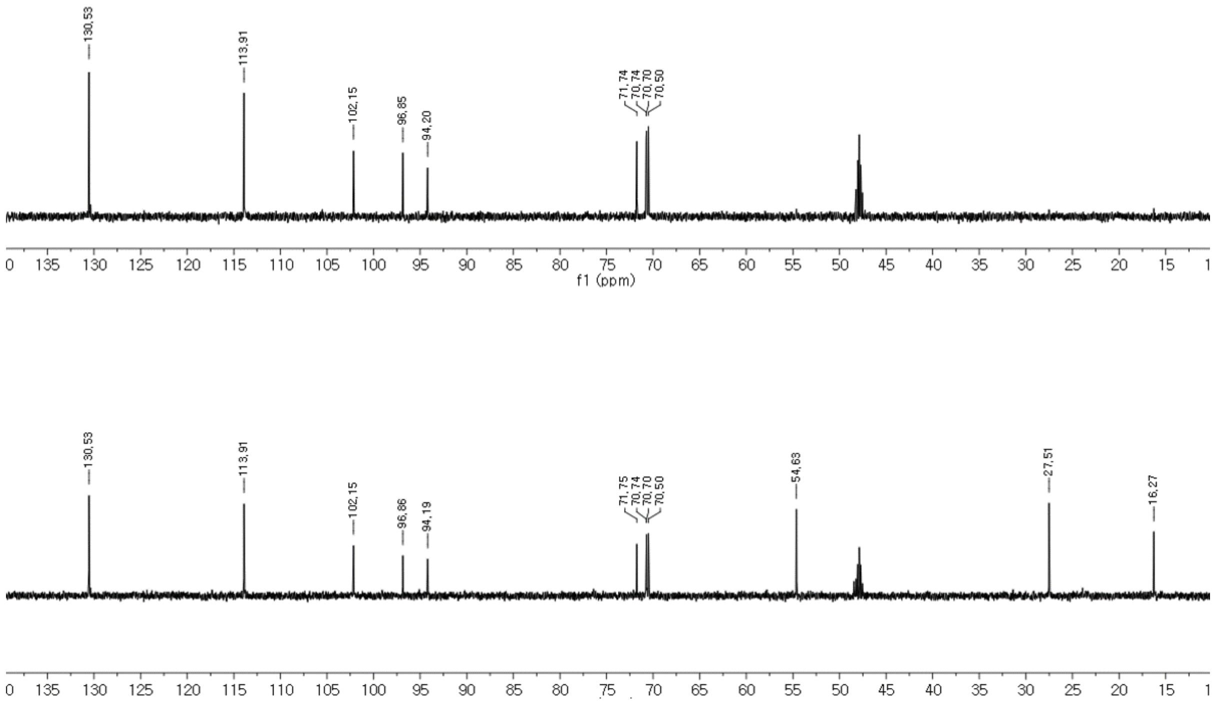

Figure 10. DEPT-90 and -135 spectrum of compound **8** (500 MHz, MeOD).

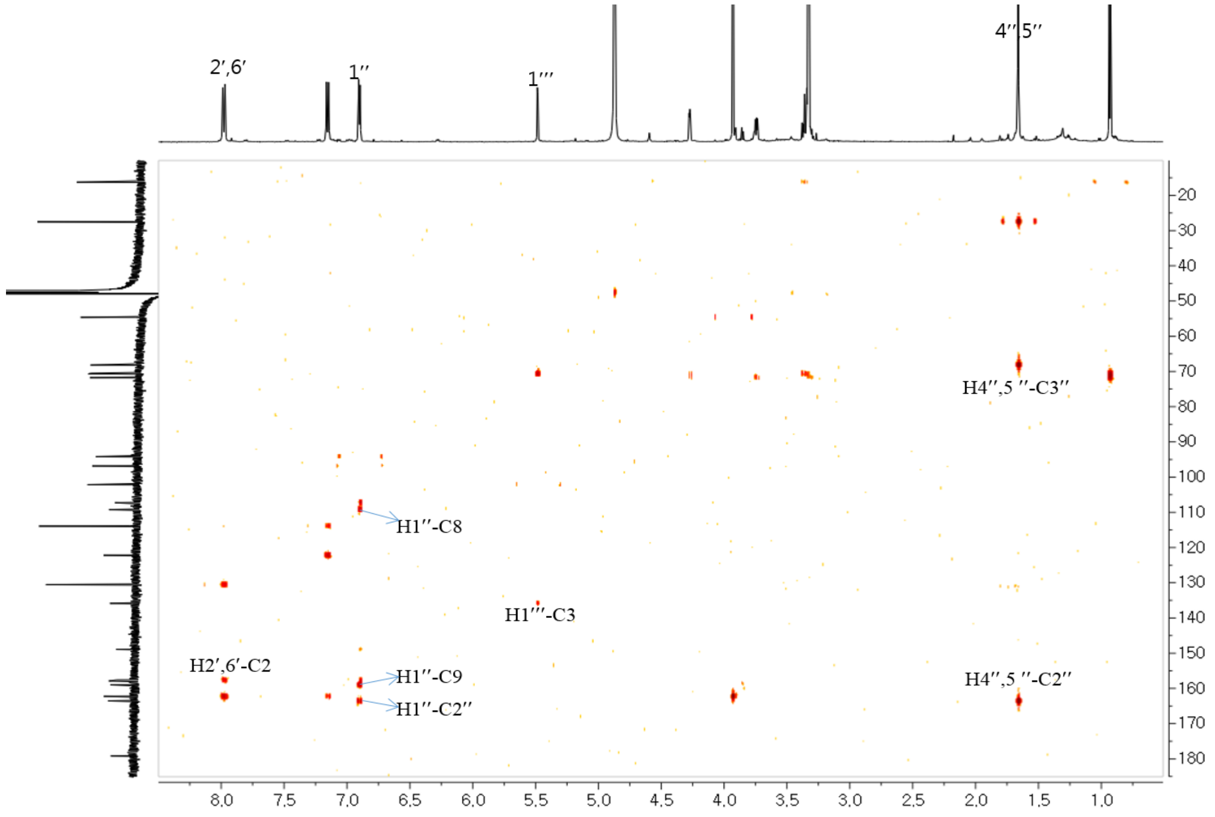

Figure 11. HMBC spectrum of compound **8** (500 MHz, MeOD).

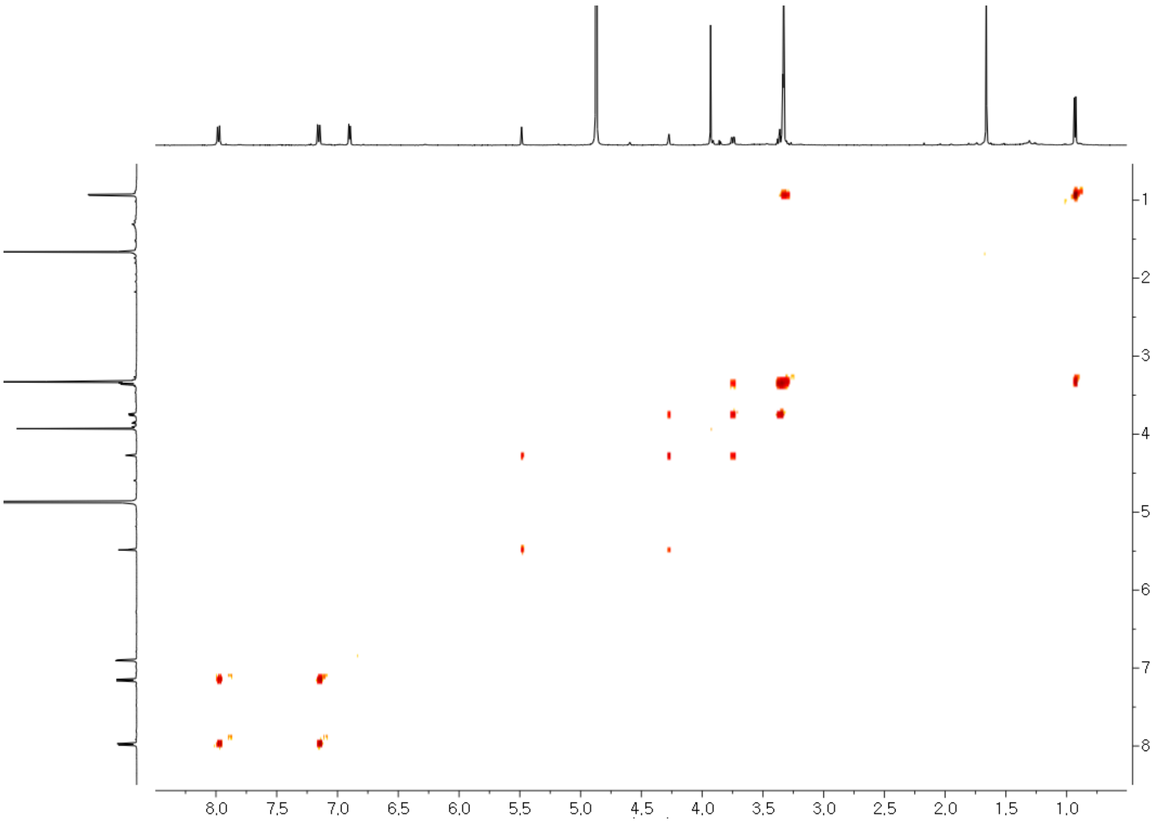

Figure 12. COSY spectrum of compound **8** (500 MHz, MeOD).

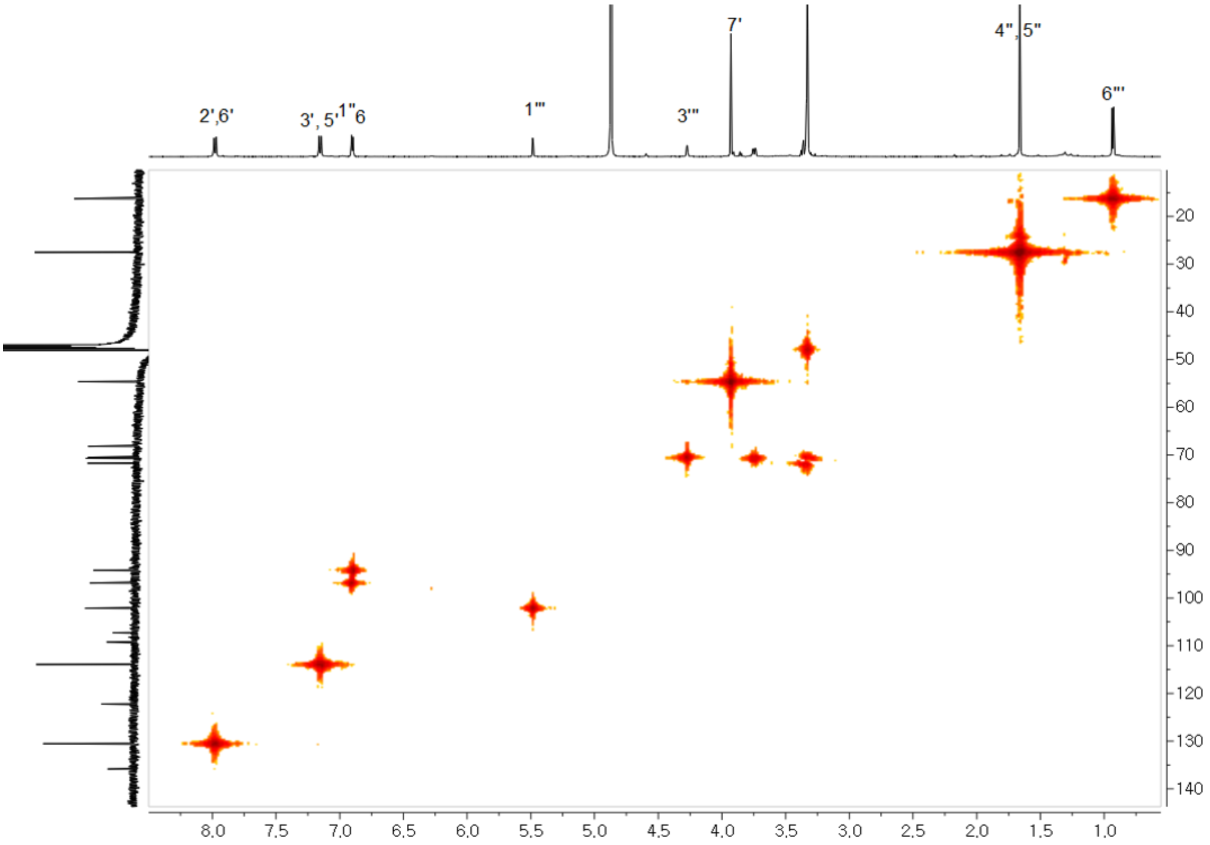

Figure 13. HMQC spectrum of compound **8** (500 MHz, MeOD).

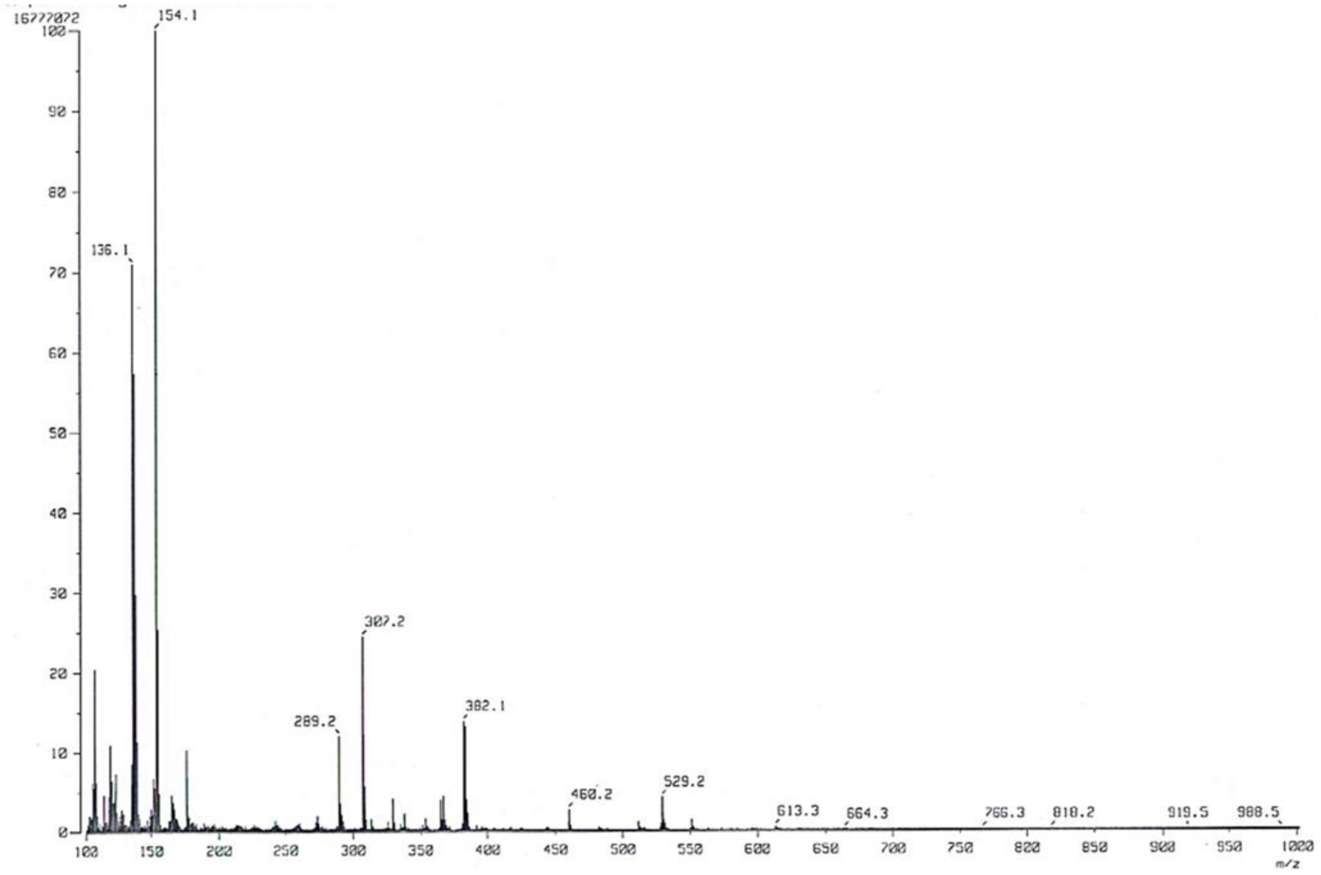


```
[ Elemental Composition ]
Data   : HM-8-HR1                          Date : 05-Apr-2018 17:02
Sample: -
Note : -
Inlet : Direct                             Ion Mode : FAB+
RT : 4.75 min                              Scan#: 58
Elements : C 100/1, H 100/1, O 20/1
Mass Tolerance      : 100ppm, 10mmu if m/z > 100
Unsaturation (U.S.) : 0.0 - 20.0

Observed m/z  Int%    Err[ppm / mmu]     U.S.  Composition
  529.1682    19.9        -5.2 /  -2.8    13.5  C 27 H 29 O 11
                         -16.3 /  -8.6     4.5  C 20 H 33 O 16
                         +12.5 /  +6.6     0.5  C 16 H 33 O 19
```

Figure 14. FABMS spectra and HRFABMS data of compound **8**.

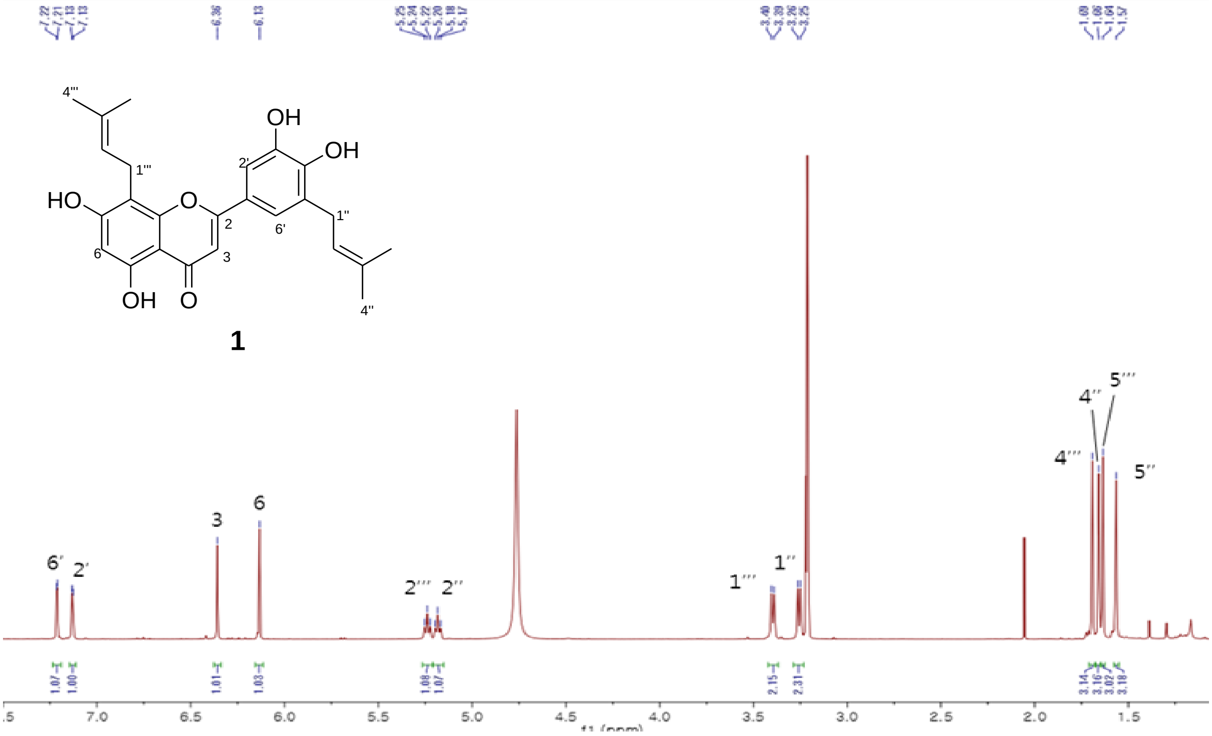

Figure 15. ^{1}H-NMR spectrum of compound **1** (500 MHz, MeOD)

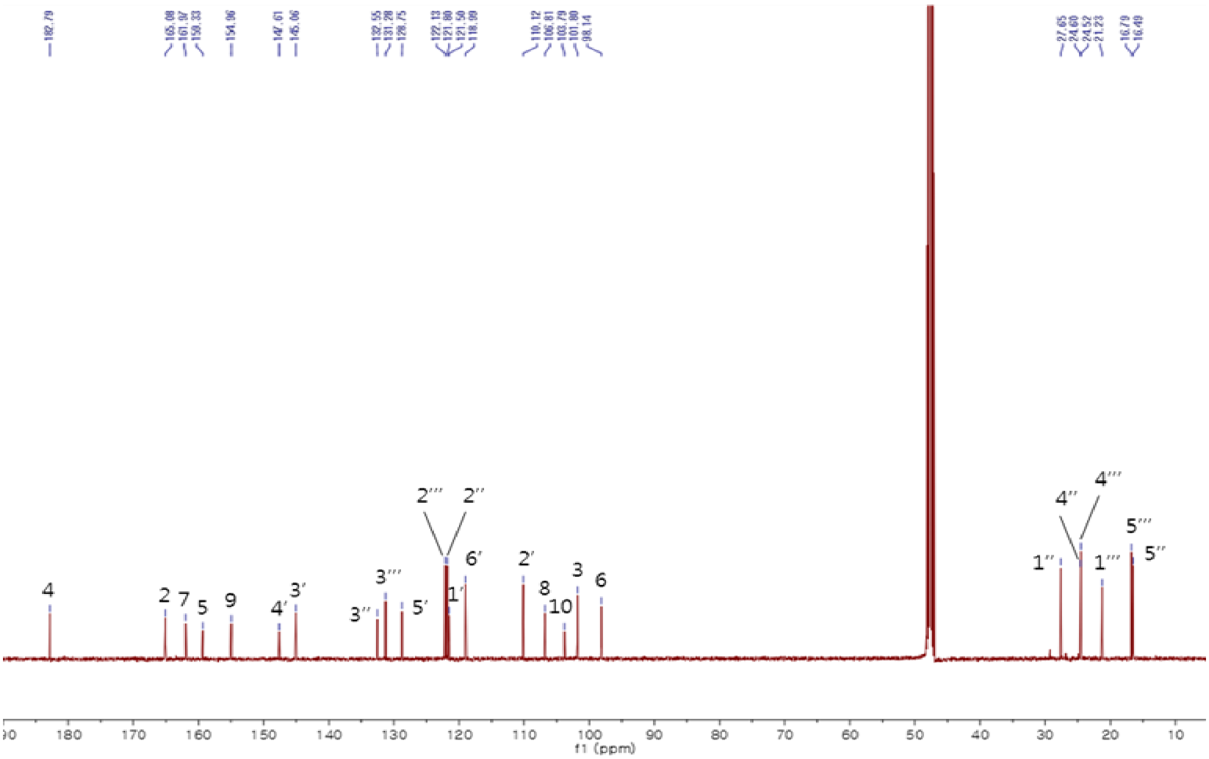

Figure 16. ^{13}C-NMR spectrum of compound **1** (500 MHz, MeOD)

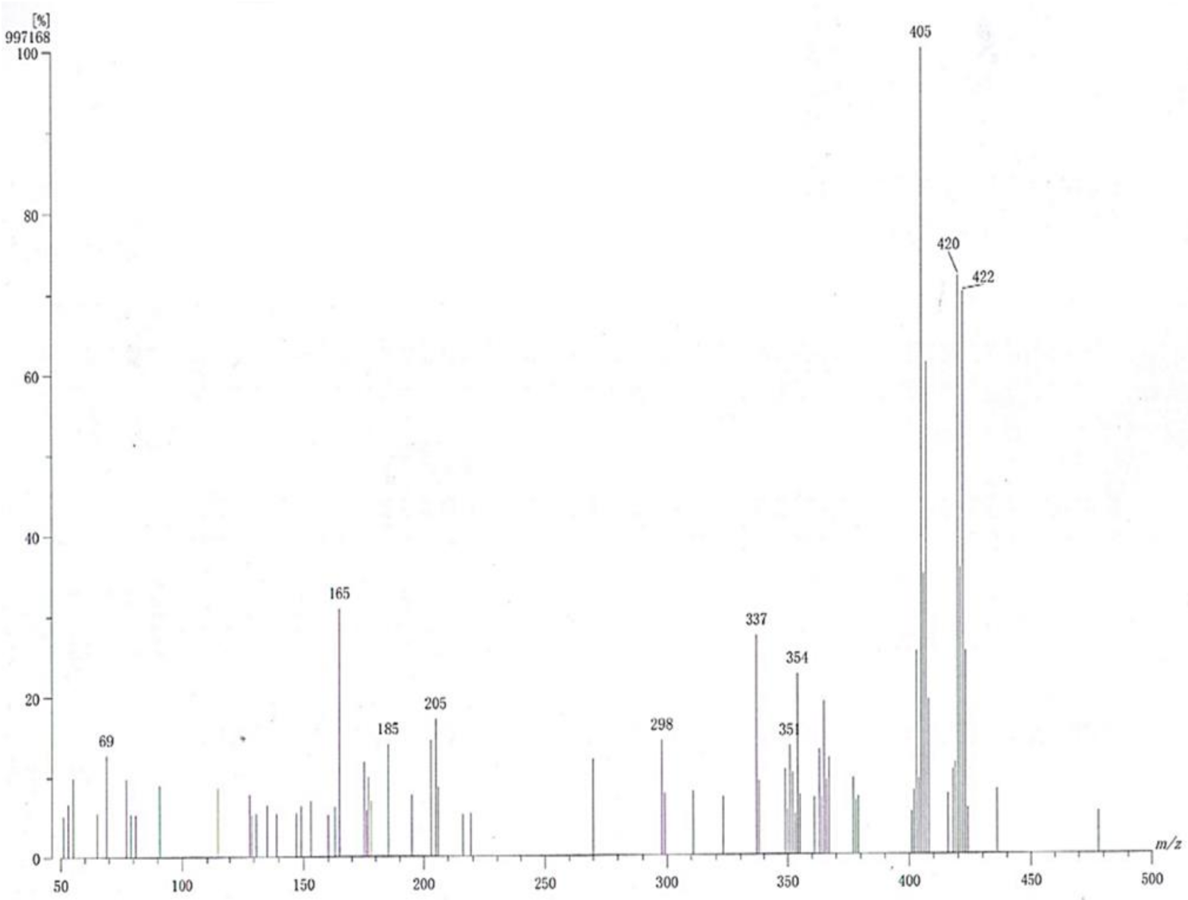

Data : HM-1-HR Date : 20-Mar-2018 15:43
Instrument : MStation
Sample : -
Note : -
Inlet : Direct Ion Mode : EI+
RT : 1.34 min Scan# : 21
Elements : C 100/1, H 100/1, O 10/1
Mass Tolerance : 1000ppm, 3mmu if m/z > 3
Unsaturation (U.S.) : -0.5 - 20.0

	Observed m/z	Int%	Err[ppm / mmu]	U.S.	Composition
1	422.1721	27.76	-2.0 / -0.8	13.0	C25 H26 O6

Figure 17. EIMS spectrum and HREIMS data of compound **1**

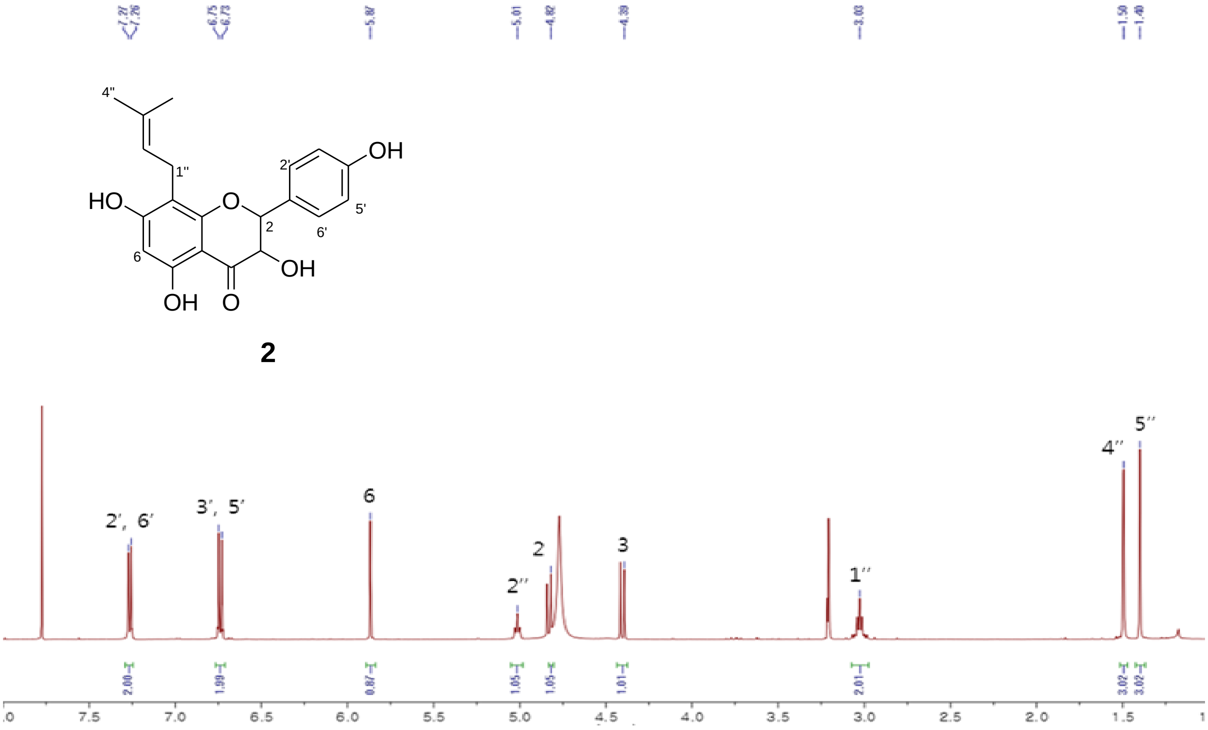

Figure 18. ^{1}H-NMR spectrum of compound **2** (500 MHz, MeOD)

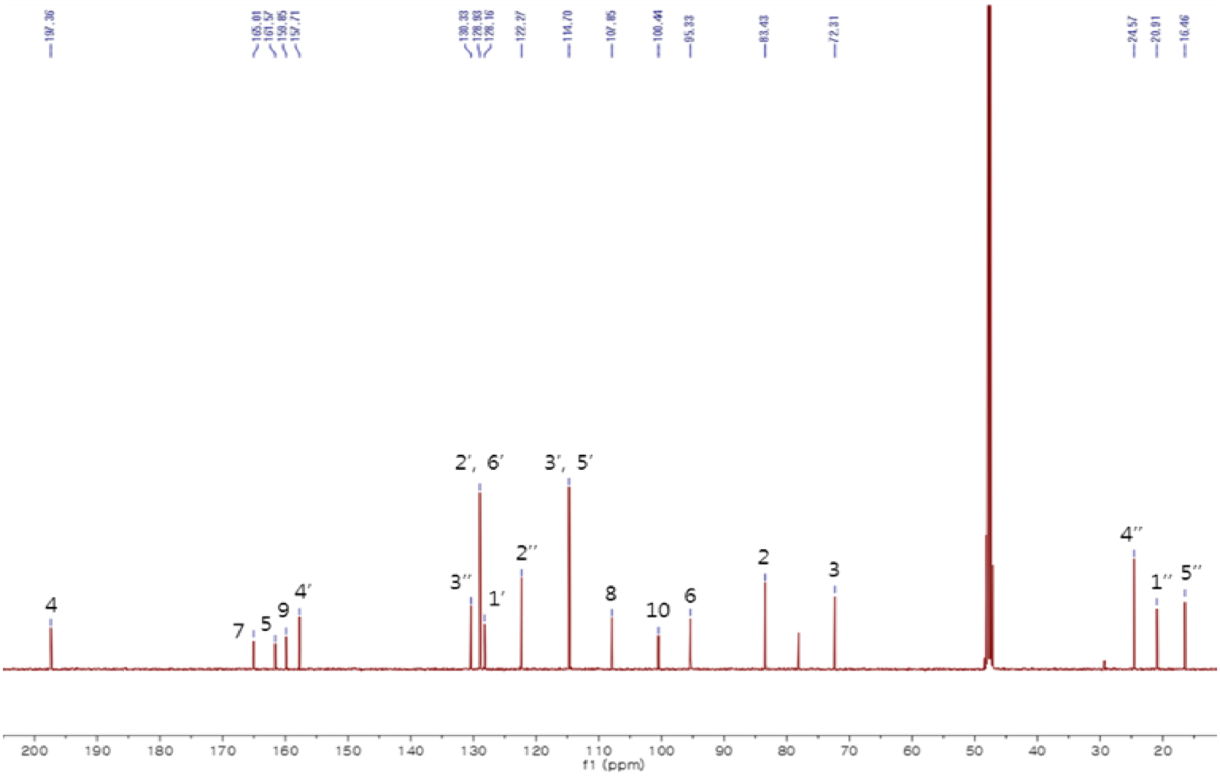

Figure 19. ^{13}C-NMR spectrum of compound **2** (500 MHz, MeOD)

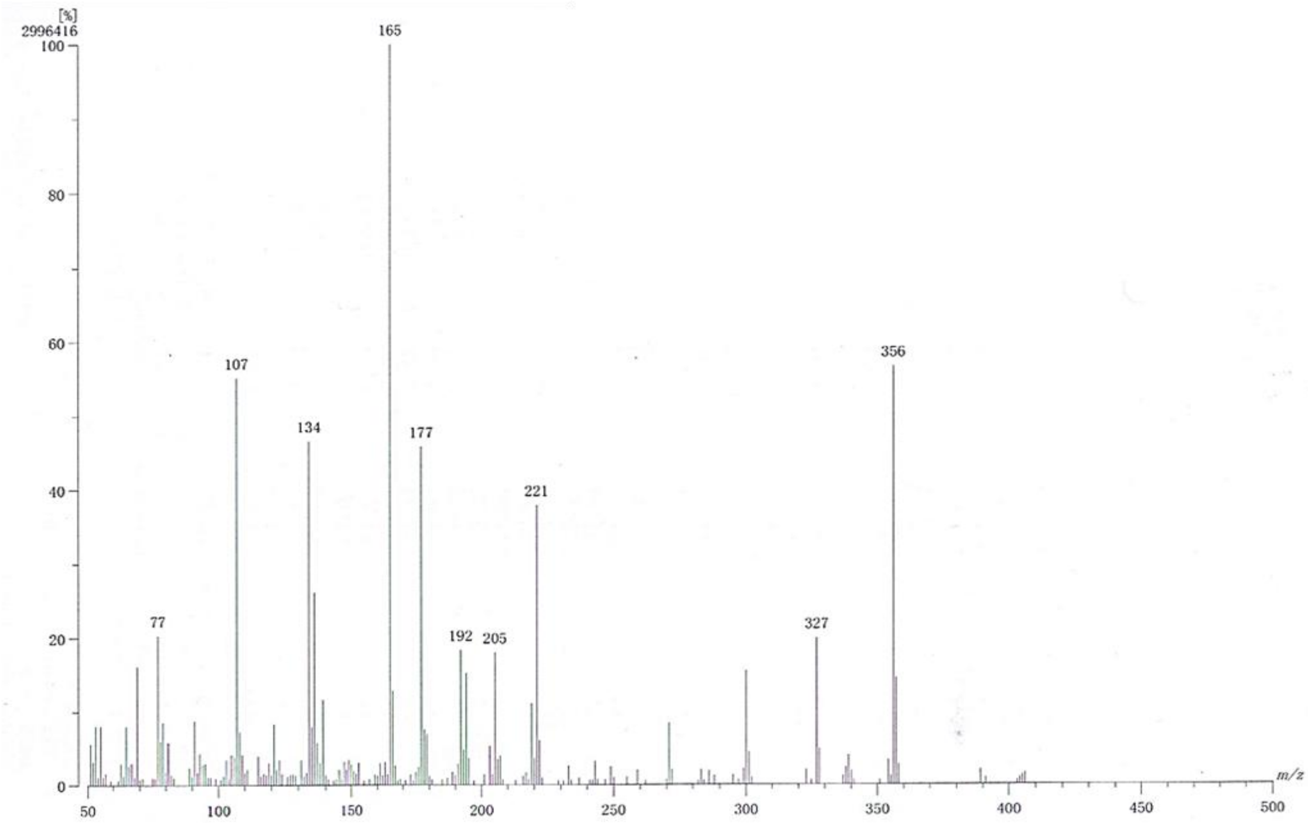

Data : HM-2-HR Date : 20-Mar-2018 15:31
Instrument : MStation
Sample : –
Note : –
Inlet : Direct Ion Mode : EI+
RT : 2.14 min Scan# : 33
Elements : C 100/1, H 100/1, O 10/1
Mass Tolerance : 1000ppm, 5mmu if m/z > 5
Unsaturation (U.S.) : -0.5 – 30.0

	Observed m/z	Int%	Err[ppm / mmu]	U.S.	Composition
1	356.1262	35.81	+0.6 / +0.2	11.0	C20 H20 O6

Figure 20. EIMS spectrum and HREIMS data of compound **2**

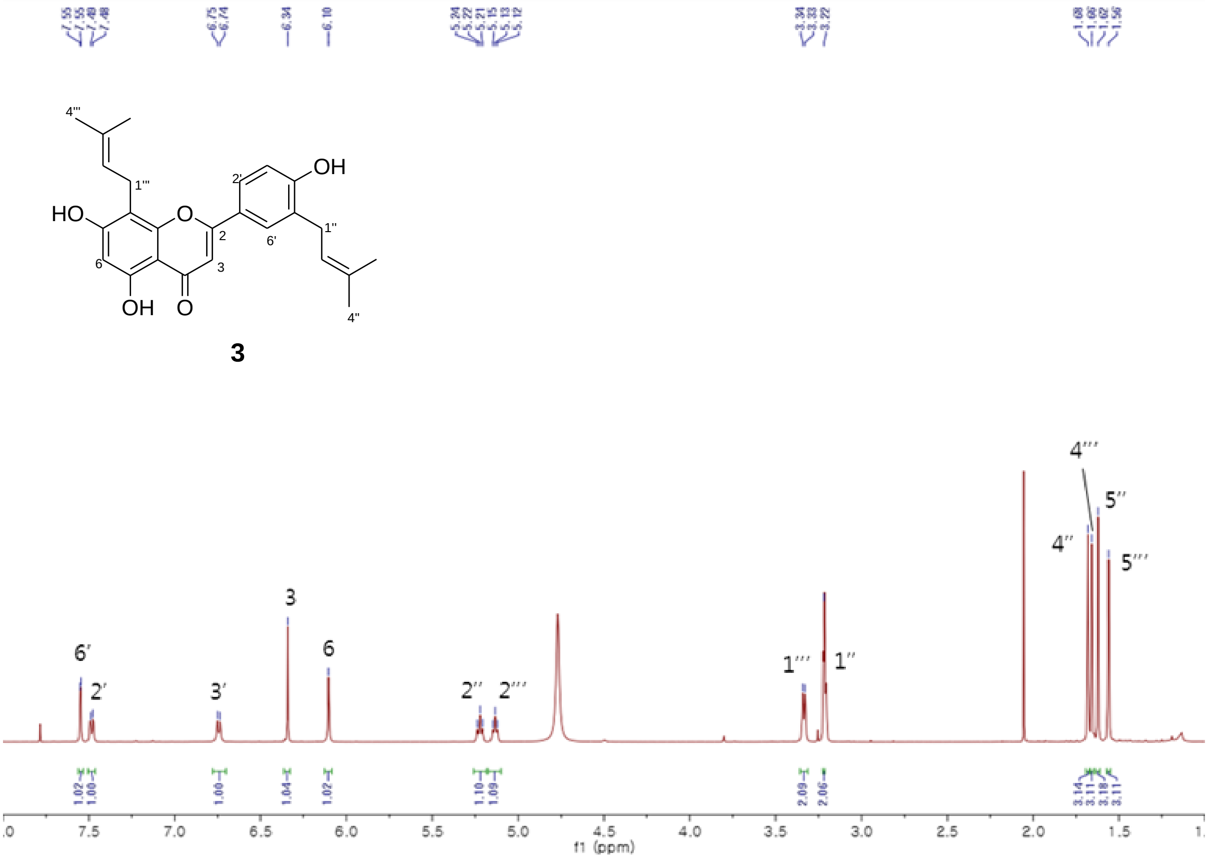

Figure 21. ^{1}H-NMR spectrum of compound **3** (500 MHz, MeOD)

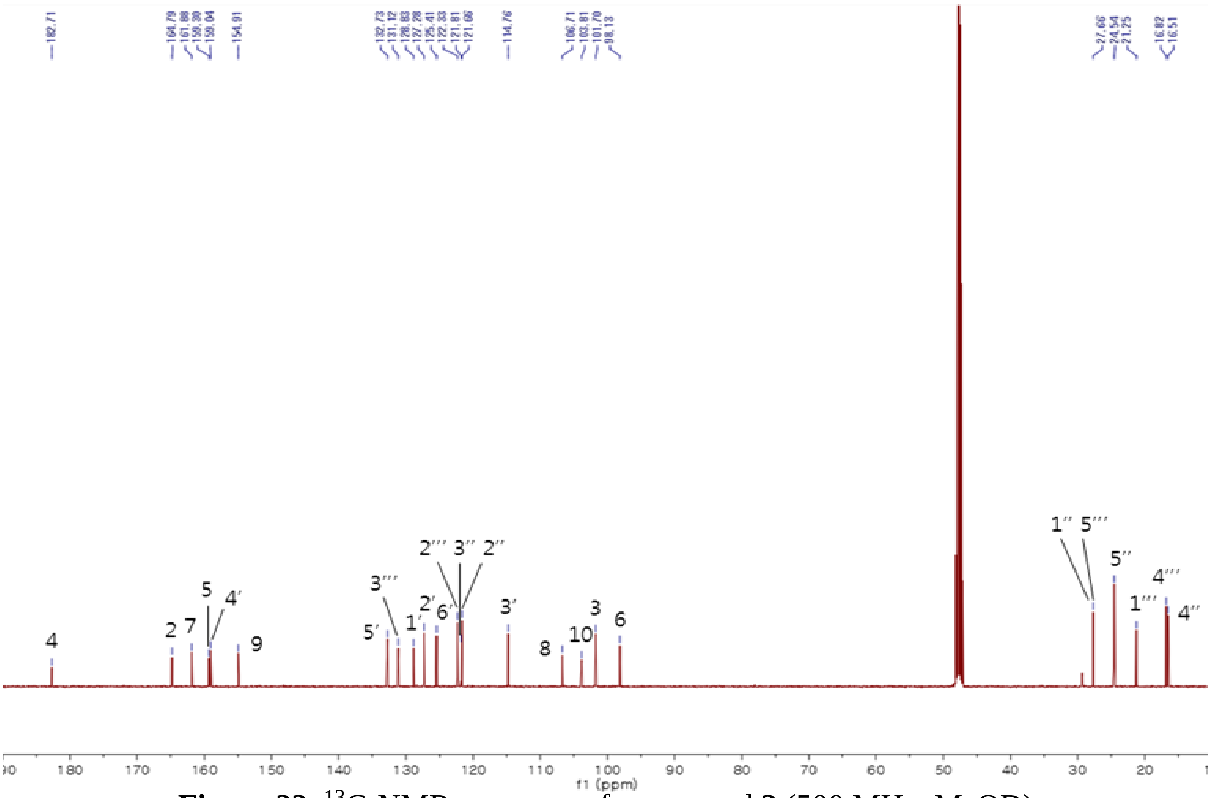

Figure 22. ^{13}C-NMR spectrum of compound **3** (500 MHz, MeOD)

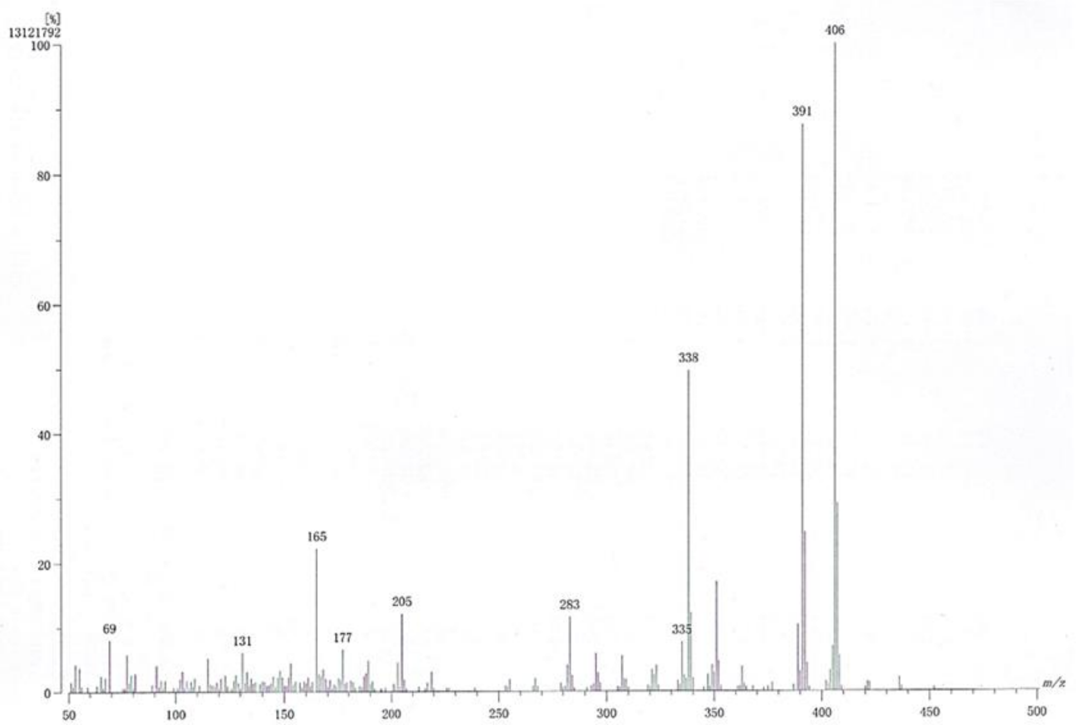

Data : HM-3-HR Date : 20-Mar-2018 15:59
Instrument : MStation
Sample : -
Note : -
Inlet : Direct Ion Mode : EI+
RT : 1.34 min Scan# : 21
Elements : C 100/1, H 100/1, O 10/1
Mass Tolerance : 1000ppm, 3mmu if m/z > 3
Unsaturation (U.S.) : -0.5 - 20.0

	Observed m/z	Int%	Err[ppm / mmu]	U.S.	Composition
1	406.1784	100.00	+0.9 / +0.4	13.0	C25 H26 O5

Figure 23. EIMS spectrum and HREIMS data of compound **3**

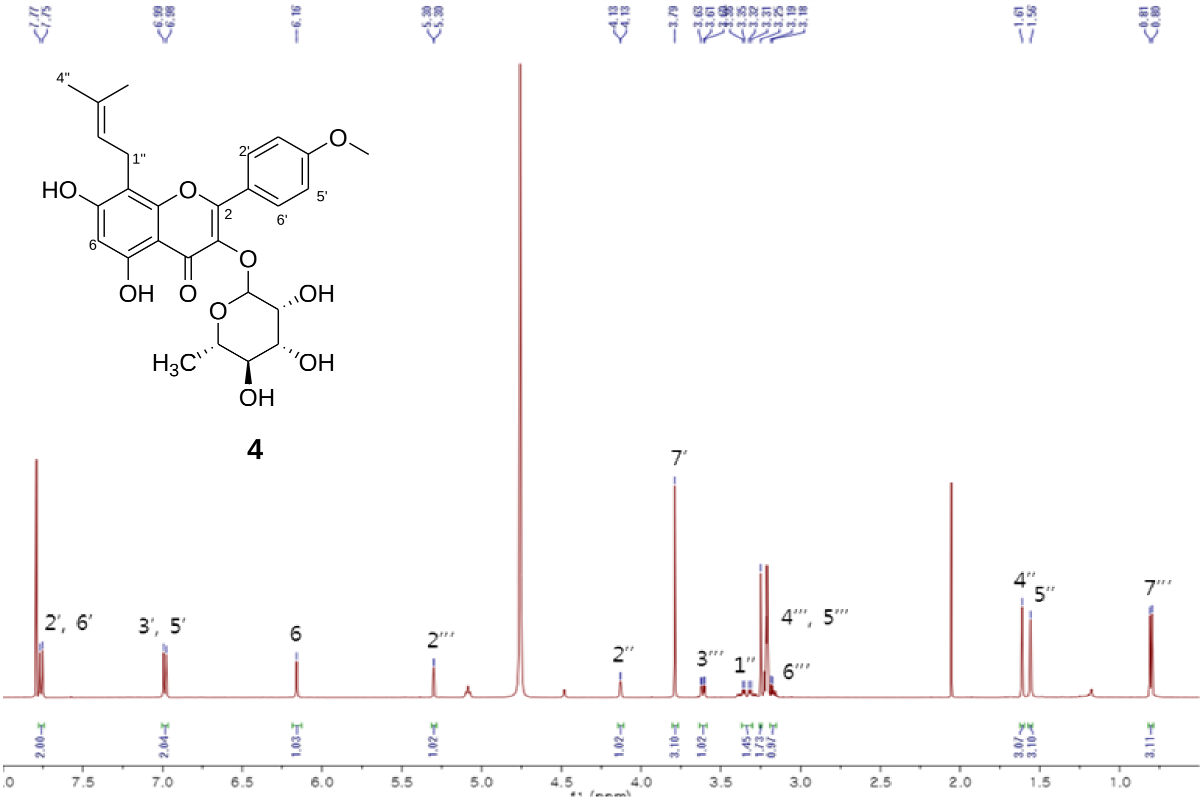

Figure 24. ^{1}H-NMR spectrum of compound **4** (500 MHz, MeOD)

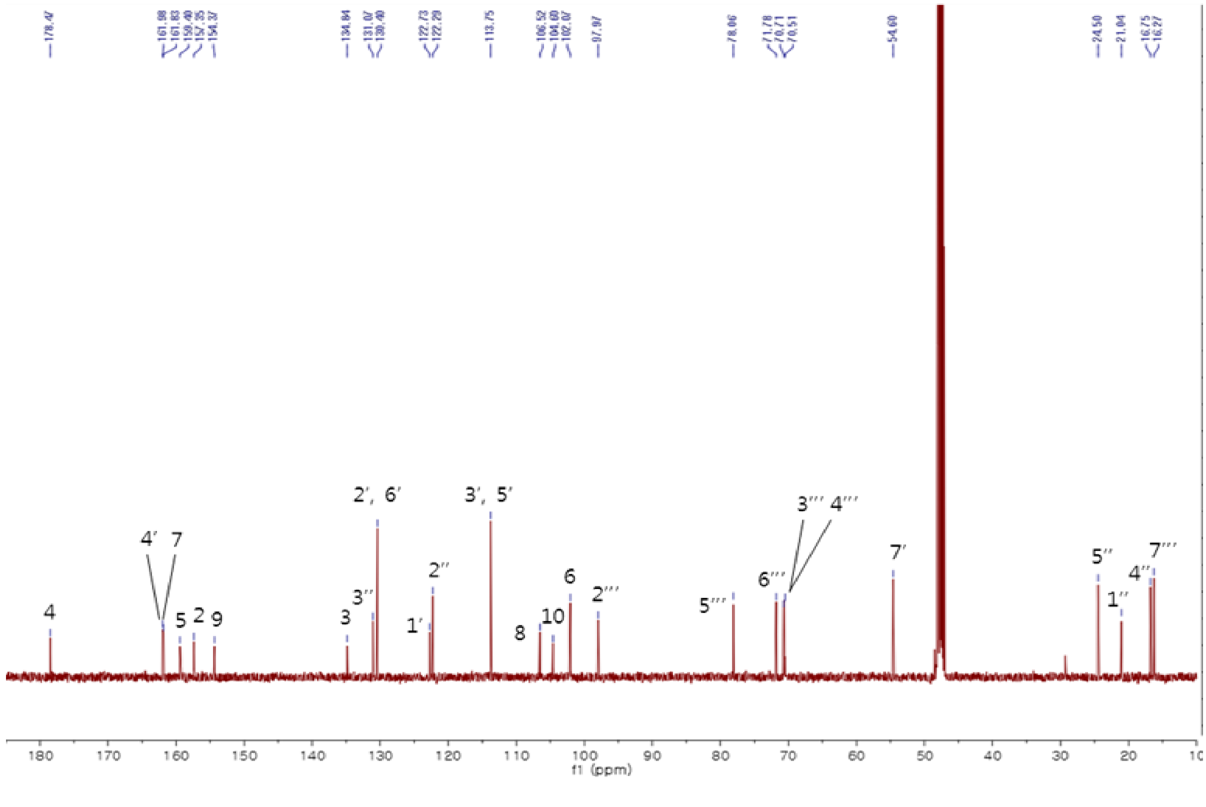

Figure 25. ^{13}C-NMR spectrum of compound **4** (500 MHz, MeOD)

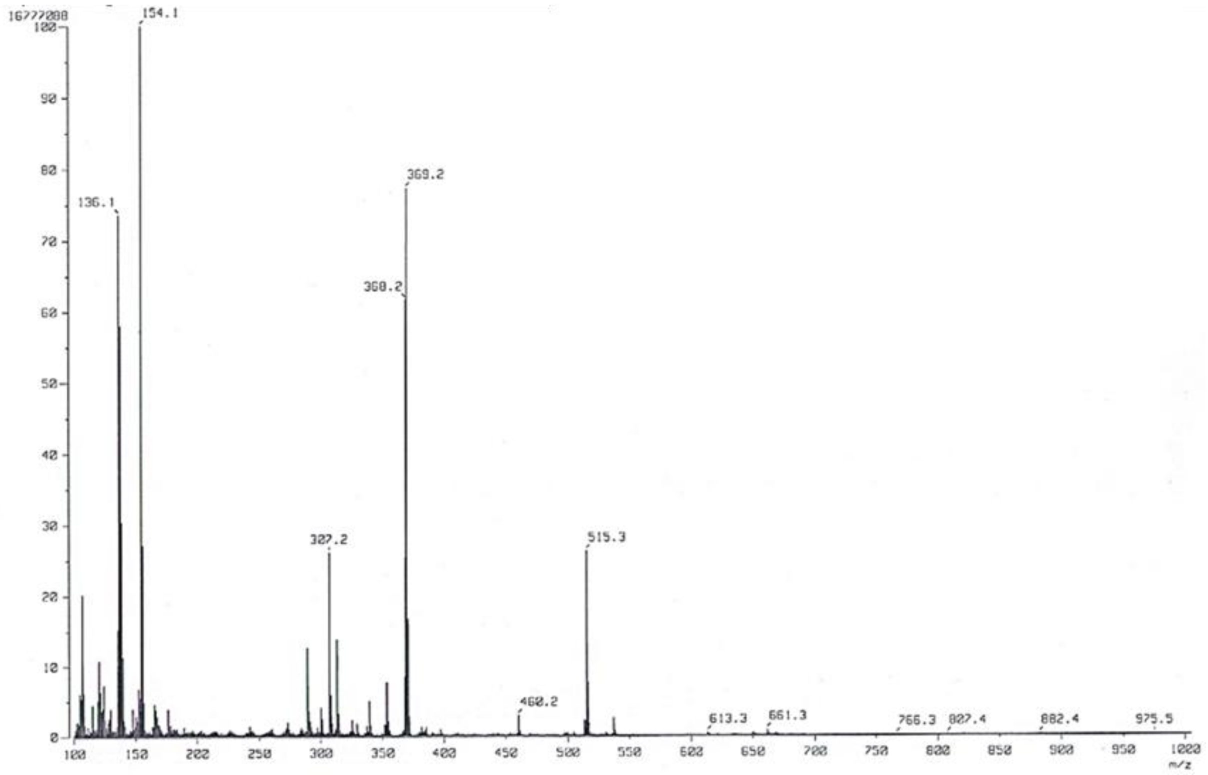

[Elemental Composition]

Data : HM4-HR1 Date : 05-Apr-2018 15:22
Sample: -
Note : -
Inlet : Direct Ion Mode : FAB+
RT : 0.84 min Scan#: 11
Elements : C 100/1, H 100/1, O 20/1
Mass Tolerance : 100ppm, 10mmu if m/z > 100
Unsaturation (U.S.) : 0.0 - 20.0

Observed m/z	Int%	Err[ppm / mmu]	U.S.	Composition
515.1893	10.2	-4.8 / -2.5	12.5	C 27 H 31 O 10
		-16.2 / -8.3	3.5	C 20 H 35 O 15

Figure 26. FABMS spectrum and HRFABMS data of compound **4**

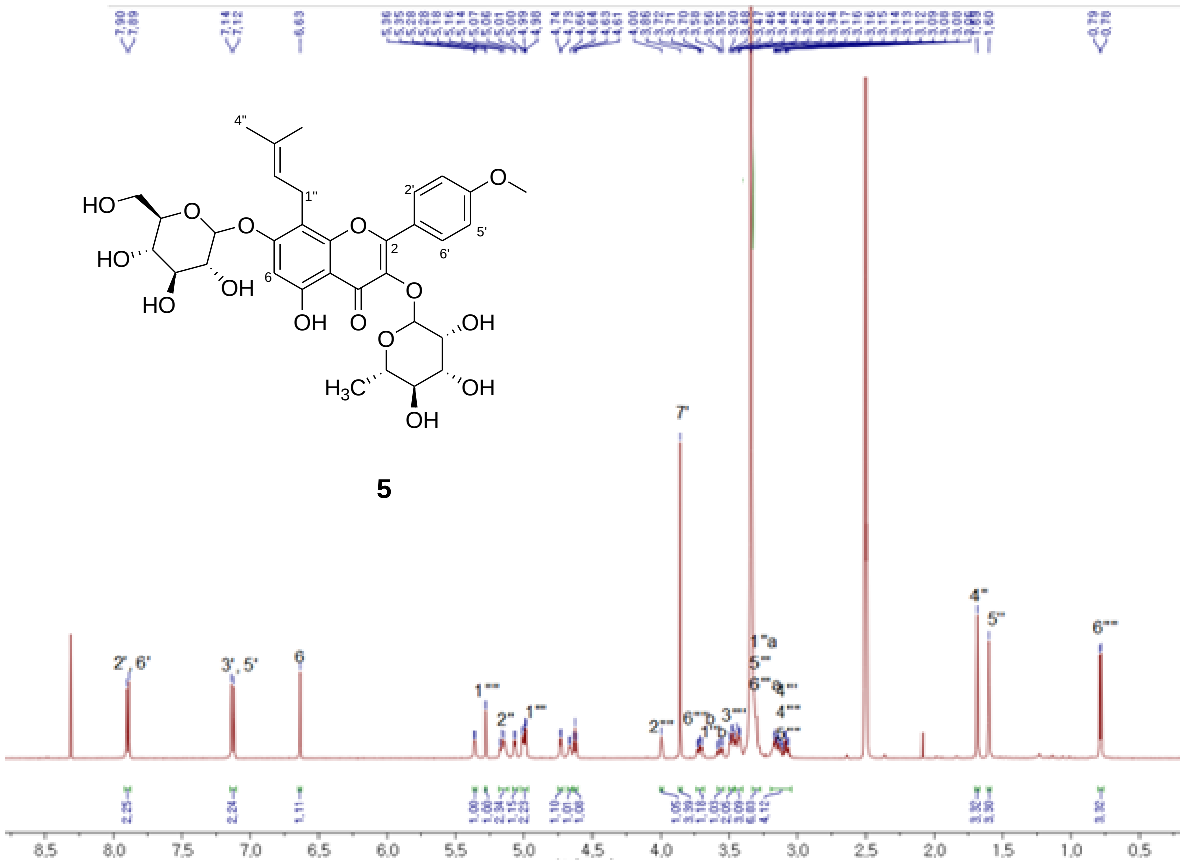

Figure 27. ^{1}H-NMR spectrum of compound **5** (500 MHz, DMSO)

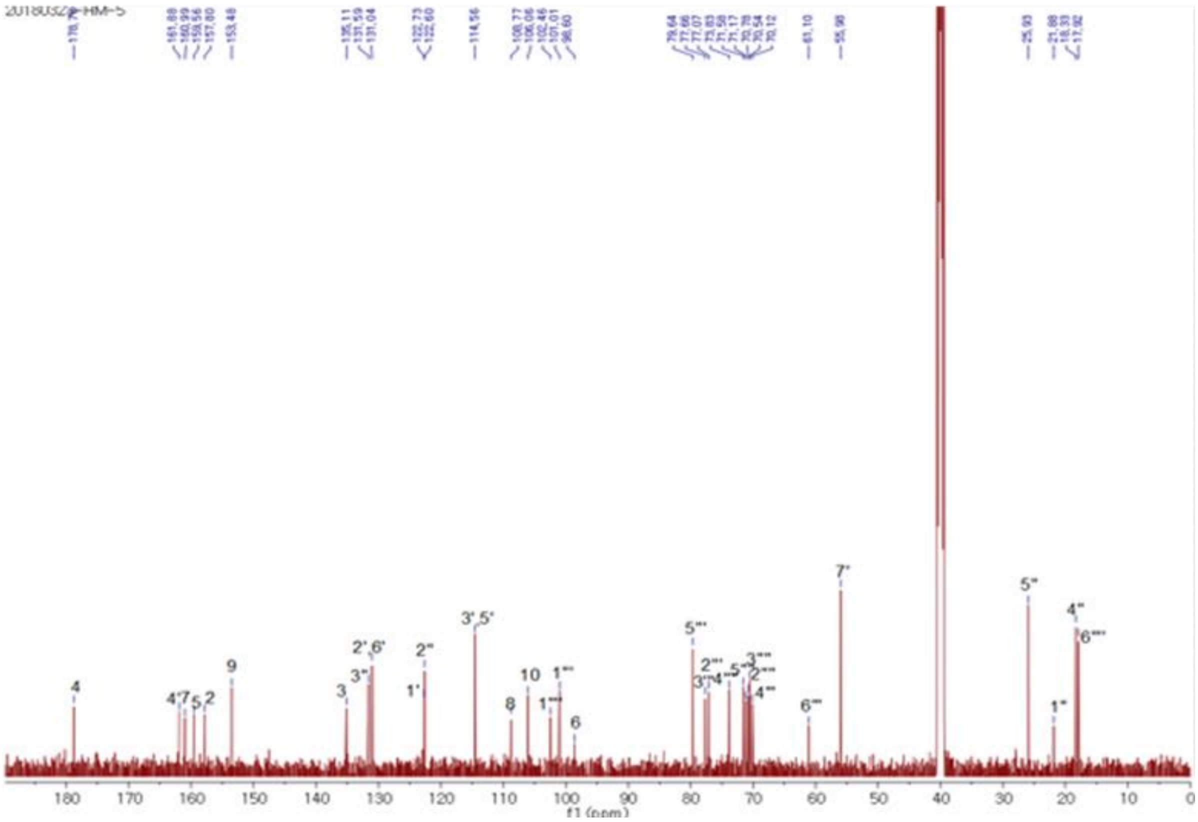

Figure 28. ^{13}C-NMR spectrum of compound **5** (500 MHz, DMSO)

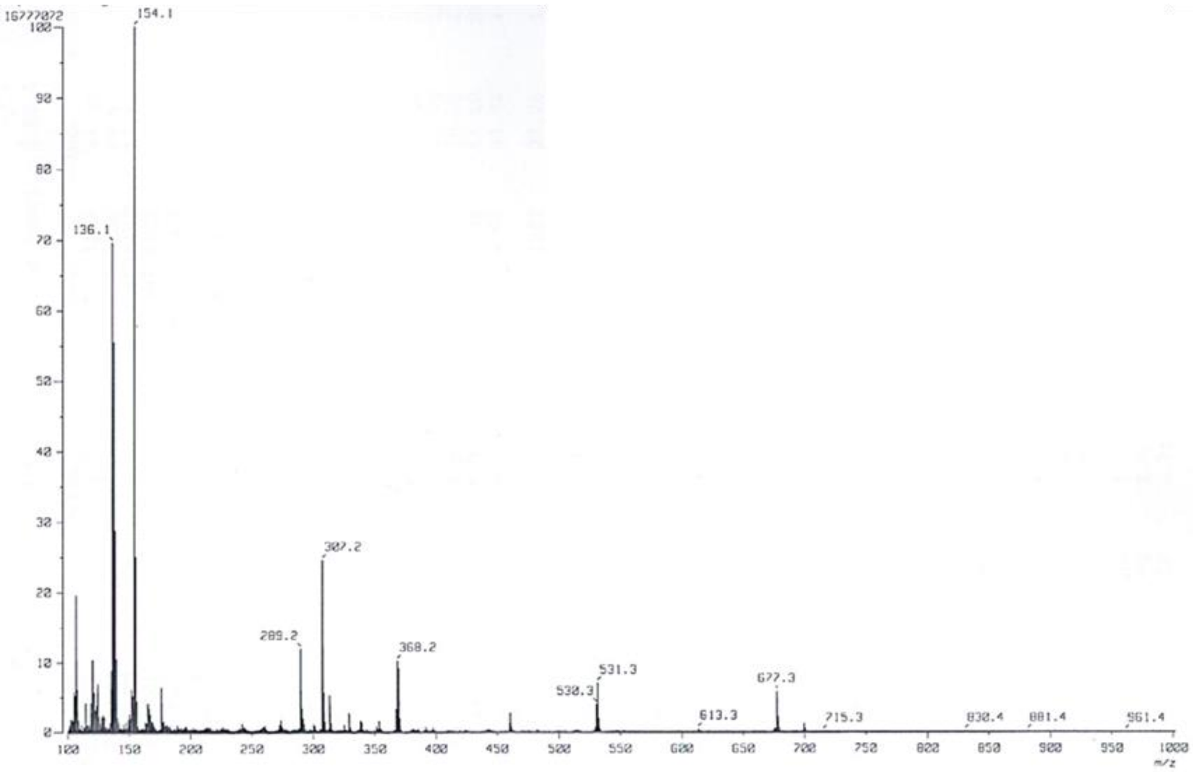

[Elemental Composition]

Data : HM-5-HR1   Date : 05-Apr-2018 15:58

Sample: -

Note : -

Inlet : Direct   Ion Mode : FAB+

RT : 1.00 min   Scan#: 13

Elements : C 100/1, H 100/1, O 20/1

Mass Tolerance : 100ppm, 10mmu if m/z > 100

Unsaturation (U.S.) : 0.0 - 20.0

Observed m/z	Int%	Err[ppm / mmu]	U.S.	Composition
677.2460	20.1	+2.2 / +1.5	13.5	C 33 H 41 O 15
		-6.5 / -4.4	4.5	C 26 H 45 O 20

Figure 29. FABMS spectrum and HRFABMS data of compound **5**

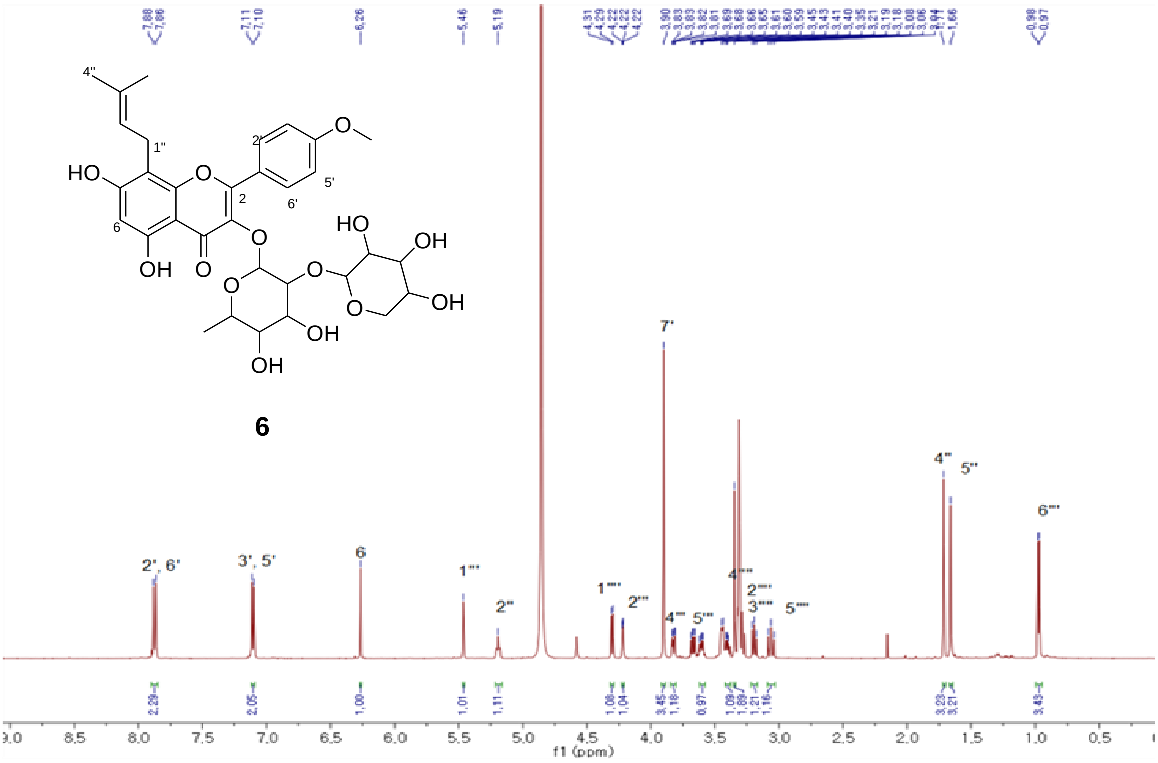

Figure 30. ^{1}H-NMR spectrum of compound **6** (500 MHz, MeOD)

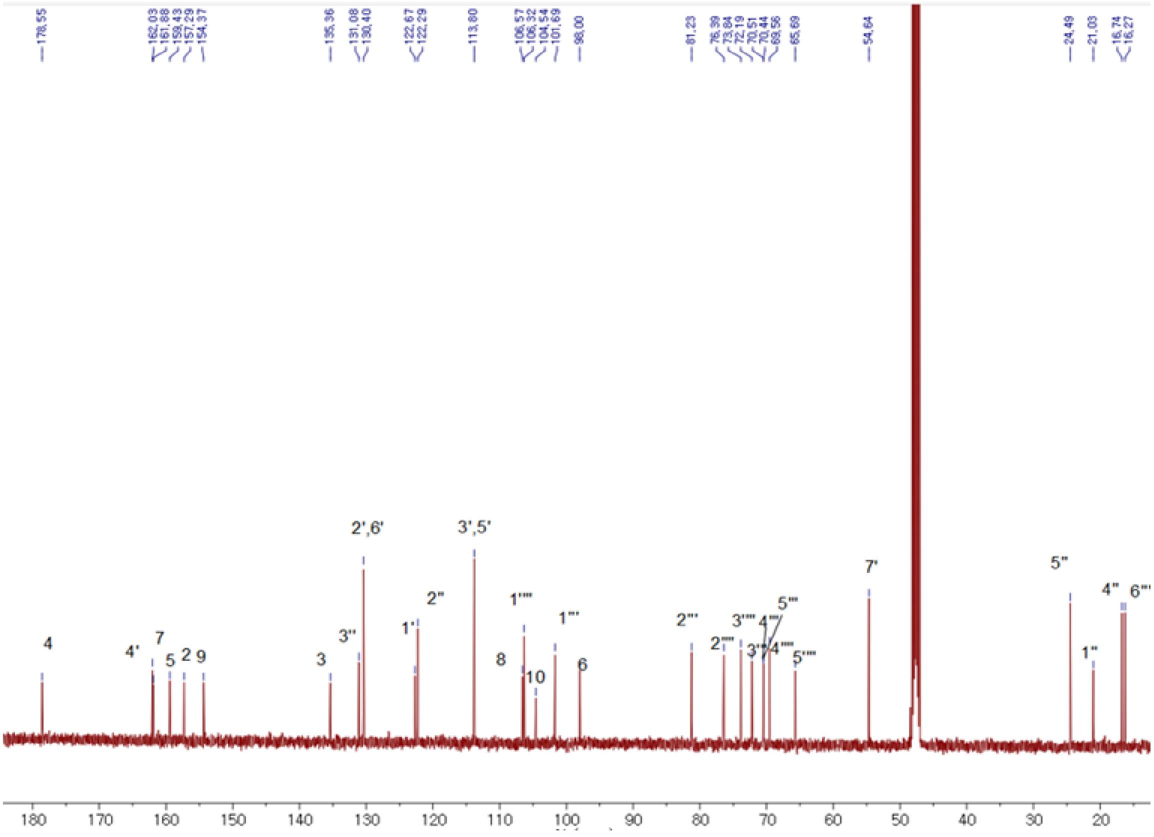

Figure 31. ^{13}C-NMR spectrum of compound **6** (500 MHz, MeOD)

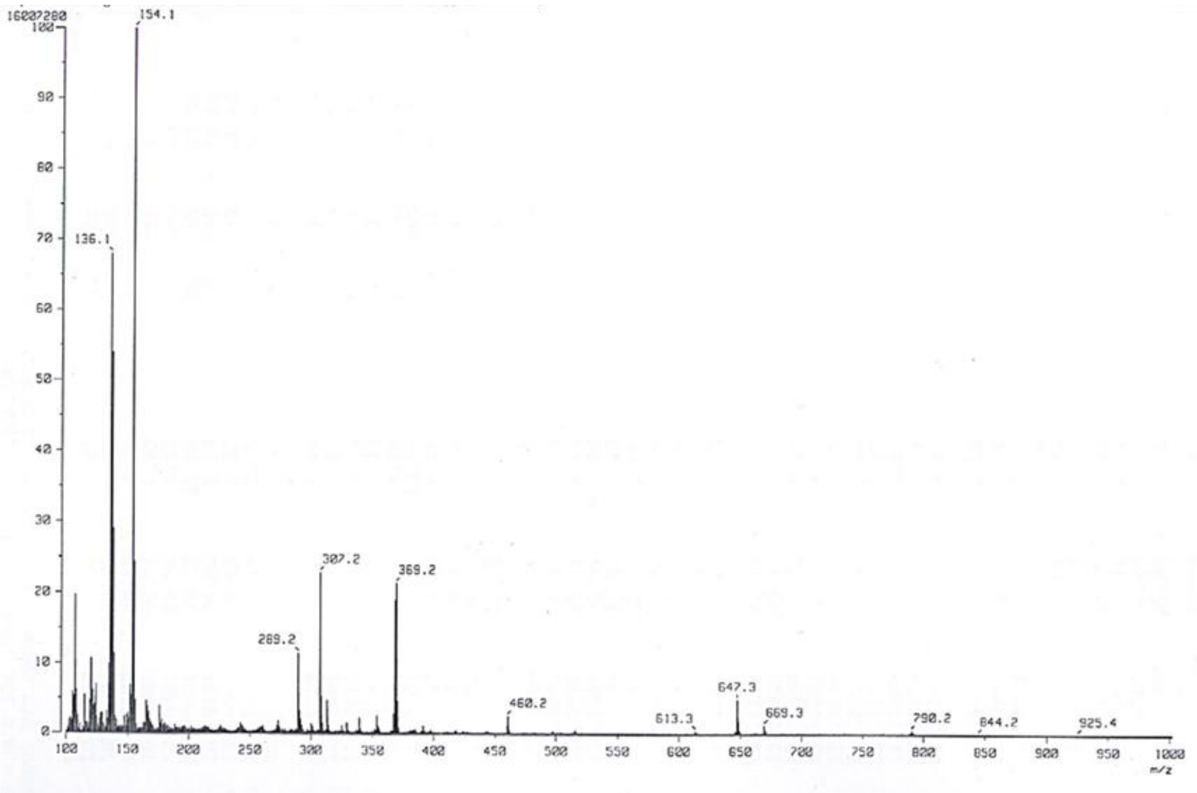

[Elemental Composition]

Data : HM-6-HR1 Date : 05-Apr-2018 16:11

Sample: -

Note : -

Inlet : Direct Ion Mode : FAB+

RT : 0.84 min Scan#: 11

Elements : C 100/1, H 100/1, O 20/1

Mass Tolerance : 100ppm, 10mmu if m/z > 100

Unsaturation (U.S.) : 0.0 - 20.0

Observed m/z	Int%	Err[ppm / mmu]	U.S.	Composition
647.2369	27.6	+4.6 / +3.0	13.5	C 32 H 39 O 14
		-4.5 / -2.9	4.5	C 25 H 43 O 19

Figure 32. FABMS spectrum and HRFABMS data of compound **6**

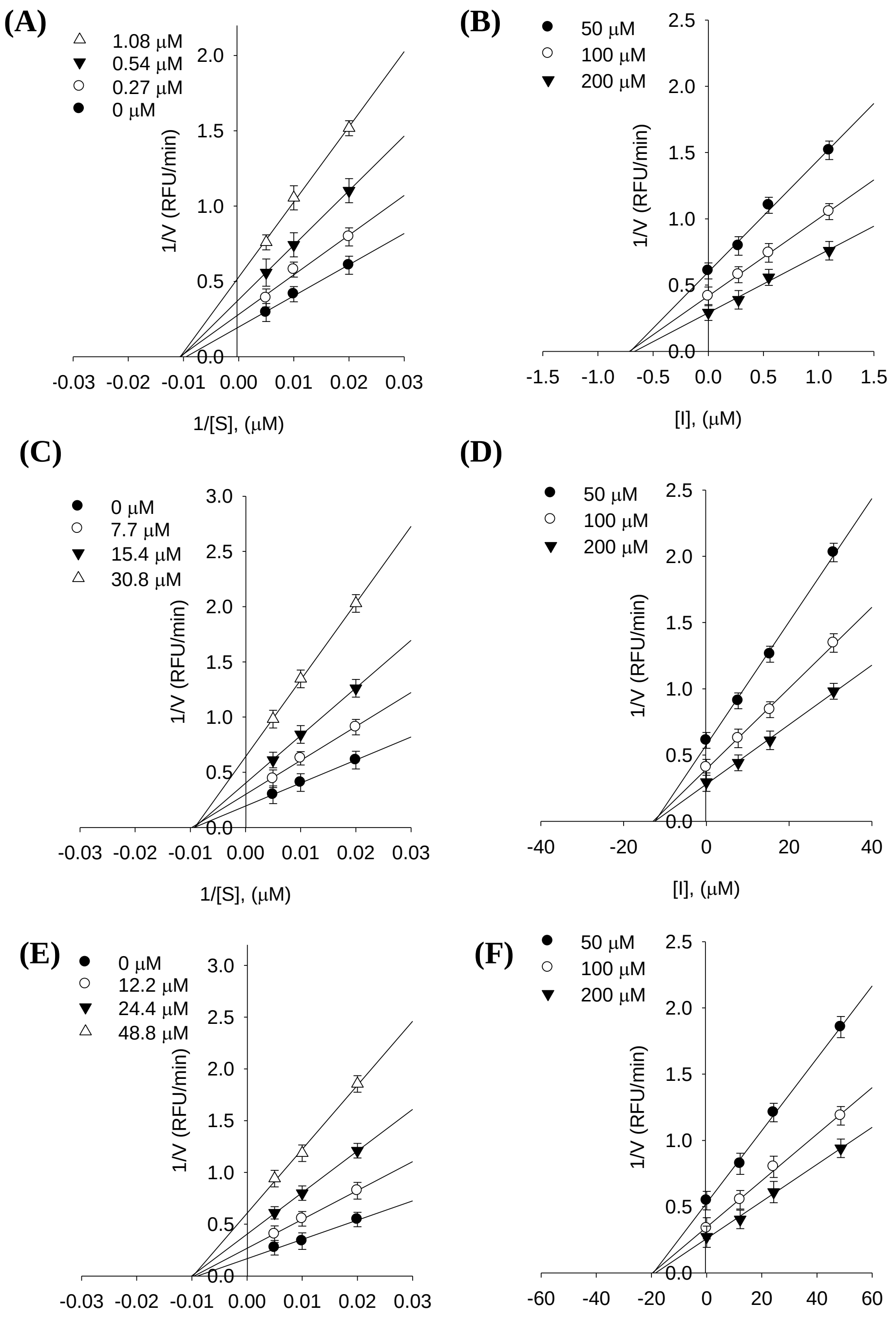

Figure 33. (A, C, E) Lineweaver-Burk plots of compounds **2-4** on bacterial neuraminidase inhibition, (B, D, F) Dixon plots of compounds **2-4** on bacterial neuraminidase inhibition.

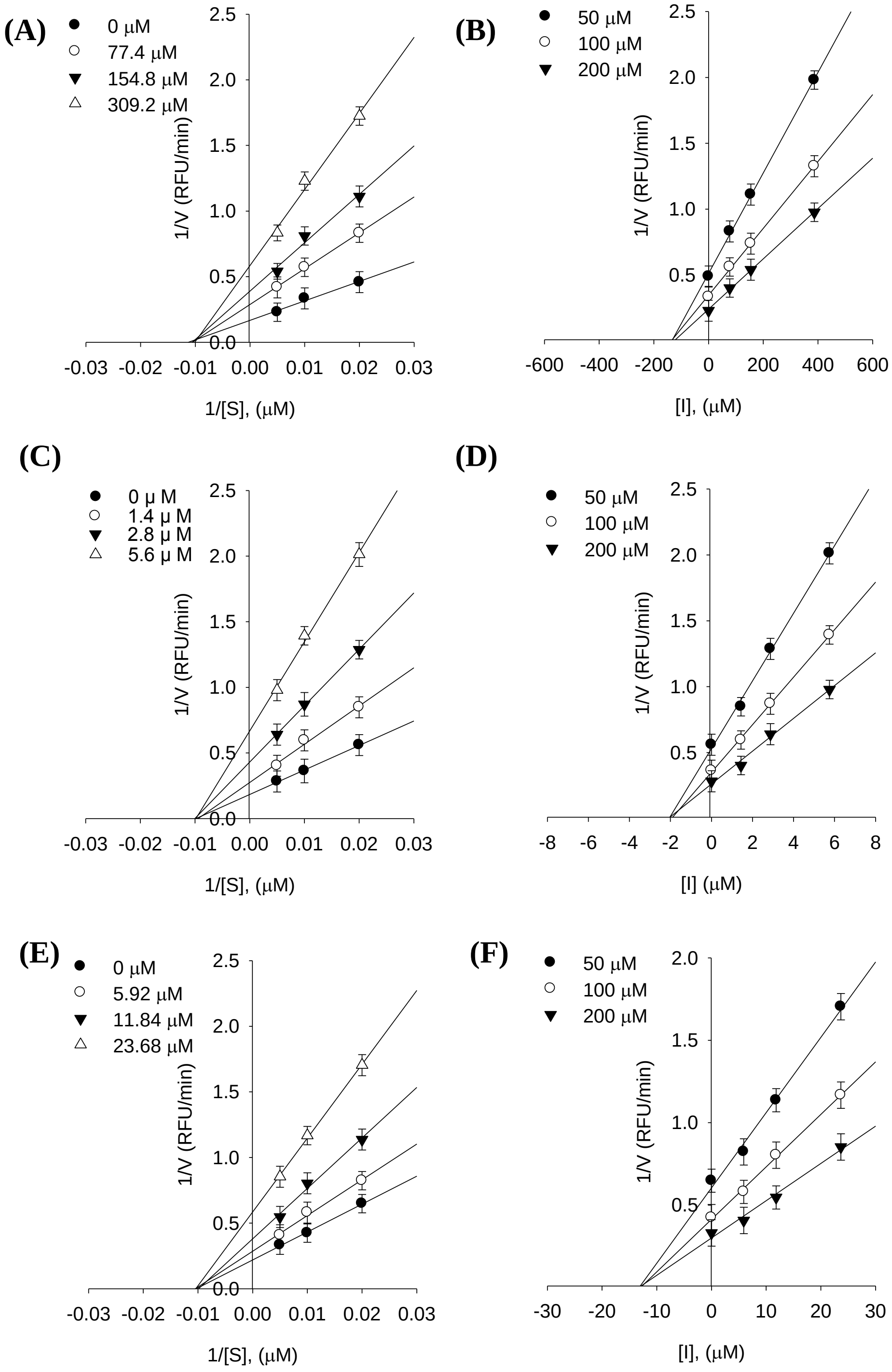

Figure 34. (A, C, E) Lineweaver-Burk plots of compounds **6-8** on bacterial neuraminidase inhibition, (B, D, F) Dixon plots of compounds **6-8** on bacterial neuraminidase inhibition.

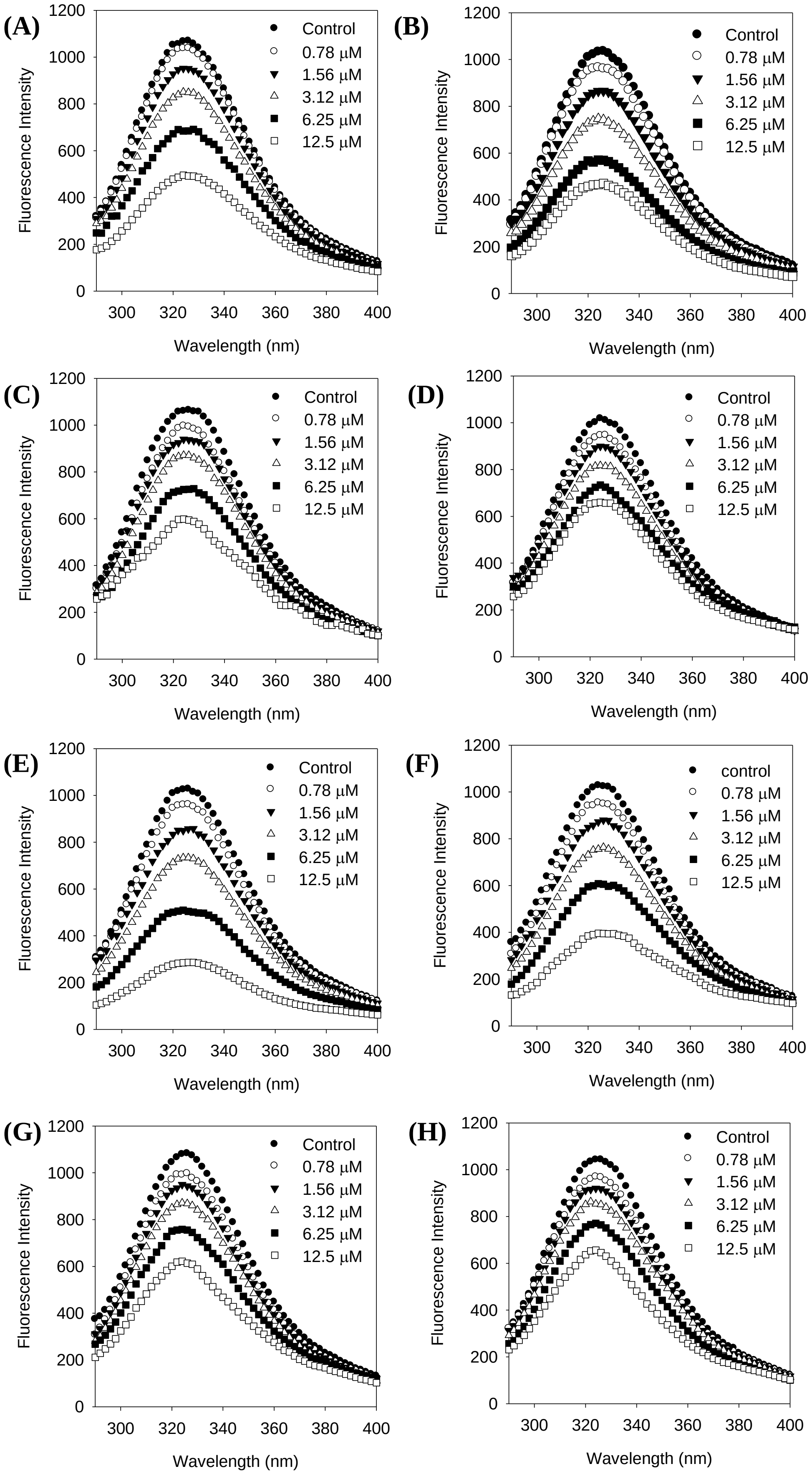

Figure 35. Fluorescence quenching effect of (A-H) **3-8**, luteolin, and apigenin, respectively.
